# Synthesis and biological screening of new thiadiazolopyrimidine-based polycyclic compounds

**DOI:** 10.1038/s41598-021-95241-x

**Published:** 2021-08-03

**Authors:** Alaa M. Alqahtani

**Affiliations:** Pharmaceutical Chemistry Department, College of Pharmacy, Umm Al-Qura University, Makkah, 21955 Saudi Arabia

**Keywords:** Chemical biology, Medicinal chemistry, Organic chemistry

## Abstract

Novel tri-and tetra-cyclic compounds based on the thiadiazolopyrimidine ring system were synthesized, and their antimicrobial activity was estimated. The obtained results evidenced the substantial efficiencies of pyrano-thiadiazolopyrimidine compounds **8a–b** and **9a–b** toward the two strains of gram-positive bacteria (*S. aureus* and *B. cereus*). Besides, tetracyclic pyrazolopyrimido-thiadiazolopyrimidine derivatives **16a–b** and **17a–b** displayed prominent efficiencies toward the two strains of gram-negative bacteria (*E. coli* and *P. aeruginosa*). In addition, compounds **8a–b** and **9a–b** displayed good efficacy toward *C. albicans*. The activity of antiquorum sensing (anti-QS) inhibition of the newly synthesized thiadiazolopyrimidine-based compounds toward *C. violaceum* was tested, suggesting satisfactory activity for derivatives **16a–b**, **17a–b**, **8b,** and **9a**. The cytotoxic activity of these derivatives was screened toward various cancer cell lines (MCF-7, PC3, Hep-2, and HepG2) and standard normal fibroblast cells (WI38) by utilizing the MTT assay. The pyrazolopyrimido-thiadiazolopyrimidine derivatives **16a**, **16b17a**, and **17b** showed potent cytotoxic efficacy against the MCF-7 cells with the IC_50_ values ranging from 5.69 to 9.36 µM. Also, the endorsed structural activity relationship (SAR) of the inspected thiadiazolopyrimidine derivatives provided a correlation between the chemical structure and anticancer efficiency. The in silico docking studies were implemented for silencing the hormonal signaling in the breast (PDB Code-5NQR). The results were found to be consistent with the cytotoxic activity.

## Introduction

Inspired by the important role of antibiotics in the treatment and prevention of bacterial infections, the efficiency of the drugs is inadequate with the increase in the number of pathogens resistant to the antibiotics. The resistance to antibiotics is the main risk to public health and leads to an increase in the rate of morbidity and mortality in addition to the high cost of treatment^1^. The extensive use of antibiotics causes the accumulation of microbial resistance^2^. Thus, the current antivirulence approaches were established by genetic investigation to diagnose the virulence factors of numerous pathogens, where several methods were used to situate the pressure of the pathogens. Moreover, cancer is considered one of the primary causes of death in the world^3^. It is defined as the growth of the tumor cell through its ability to disperse through other cells in the body by a progression termed as metastasis that leads to death in most cases^4,5^. Cancer therapeutics include surgical treatment, radioactive treatment, immunotherapy, chemotherapy, etc. Chemotherapy is considered the most important step in the cancer treatment protocol. Nevertheless, the lack of selectivity of anticancer agents is the main limitation to the development of cancer medication. Thiadiazolopyrimidine derivatives are an important class of fused heterocyclic moieties with widespread biological effectiveness. The thiadiazolo-pyrimidine nucleus and its derivatives, belonging to the pseudo purine class, show interesting biological profiles, including antiviral^6^, anticancer^7,8^, antibiofilm^9^, antitumor^10^, antitubercular^11^, antiglycation^12^ and antioxidant^13^ activities. In the past few decades, these analogues were synthesized as PARP1 inhibitors^14^ and STAT3 inhibitors^15^.

A series of 6-cyano-1,3,4-thiadiazolo[3,2-*a*]pyrimidine derivatives^16^ showing a good binding mode in the active site of STAT3 enzyme inhibitors^17^ was synthesized to treat breast cancer^18^. The 2-alkanesulfinyl/alkanesulfonyl-7-methyl-5*H*-1,3,4-thiadiazolo[3,2-α]pyrimidin-5-one derivatives^19^ showed good cytotoxic activity^20^, with exceptionally strong activity for the compounds containing electrophilic substituents, such as alkyl sulfoxide or alkyl sulfone, on the 2-position. A new series of biologically active sulfonamide derivatives of thiadiazolo[3,2-*a*]pyrimidine was synthesized and investigated for their antitumor activity^21^. Some of them were tested for the in vitro and in vivo antitumor activities. Abdel Rahman and coworkers^22^ synthesized substituted thiadiazolo[3,2-*a*]pyrimidines and 1,3-disubstituted thiourea. Most of the compounds exhibited potent cytotoxic activity against the tumor cell line A549 (non-small cell lung cancer cell line)^23^ using the sulforhodamine B (SRB) standard method^24^. Recently, Nagaraju and coworkers reported the green synthesis^25^ and characterization of thiadiazolo[3,2-*a*]pyrimidines via the multi-component reaction between the chosen 2-aminothiadiazoles, aldehydes and active methylene compounds in ethanol solvent at room temperature using vanadium oxide loaded on fluorapatite as a robust and sustainable catalyst. Also, 7-oxo-7*H*-[1,3,4]thiadiazolo[3,2-*a*]pyrimidine-5-carboxylate derivatives were conveniently synthesized under mild conditions through regioselective cycloaddition reactions^26^. This observation drew attention to the synthesis of polyheterocyclic compounds containing 1,3,4-thiadiazolo [3,2-*a*]pyrimidine moiety and evaluation of their antimicrobial and cytotoxic properties.

## Results and discussion

### Chemistry

**Figure 1:**
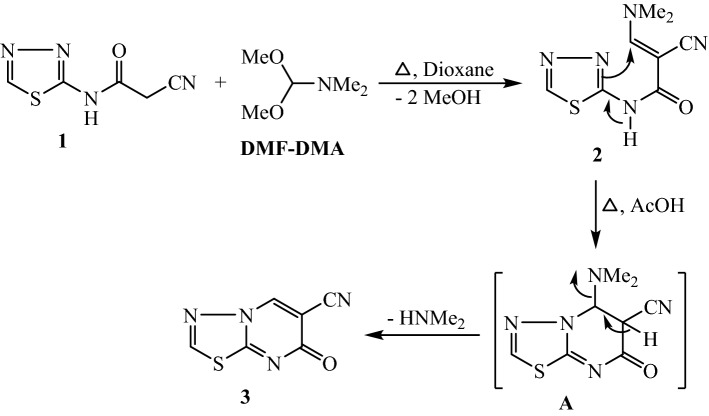
Preparation of 6-cyano-7-oxo-7*H*-[1,3,4]thiadiazolo[3,2-*a*]pyrimidine (**3**).

The reaction of 2-(2-cyanoacetamido)-1,3,4-thiadiazole **(1)**^27^ with dimethylformamide-dimethylacetal (DMF-DMA) was performed in boiling dioxane to synthesize 3-(dimethylamino)acrylonitrile derivative **2** (Fig. 1). Heating of 2-cyano-3-(dimethylamino)-*N*-(1,3,4-thiadiazol-2-yl)acrylamide **(2)** in acetic acid afforded the building block, 6-cyano-7-oxo-7*H*-[1,3,4]thiadiazolo[3,2-*a*]pyrimidine **(3)** which formed by loss of dimethylamine molecule from the intermediate **A**. The structure of **3** was supported by the spectral analyses described in the experimental section. The absorptions of nitrile and cyclic carbonyl functions were recorded in the IR spectrum at 2229 and 1690 cm^−1^, respectively. In ^1^H NMR spectrum, the proton of pyrimidine ring was observed as singlet at deshielded field, δ 9.37 ppm. The mass analysis recorded the molecular ion peak at m/z = 178, which confirmed the molecular formula C_6_H_2_N_4_OS.

**Figure 2:**
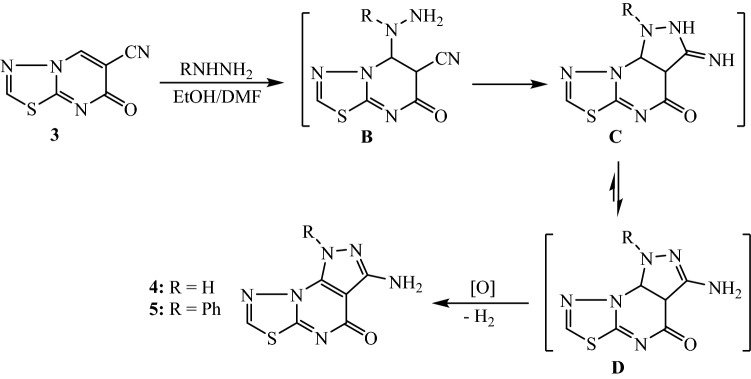
Preparation of 3-aminopyrazolothiadiazolo[3,2-*a*]pyrimidines **4** and **5**.

The thiadiazolo[3,2-*a*]pyrimidine derivative **3** was employed as a building unit for the construction of various functionalized tri- and tetra-cyclic compounds via reaction with nitrogen and carbon nucleophiles. The cyclization of thiadiazolopyrimidine **3** with hydrazine hydrate and/or phenylhydrazine was achieved by refluxing in EtOH/DMF mixture to produce the corresponding tricyclic compounds 3-aminopyrazolo[4,3-*e*]thiadiazolo[3,2-*a*]pyrimidin-4-ones **4** and **5**, respectively (Fig. 2). The chemical structures of **4** and **5** were characterized by IR, ^1^H NMR, ^13^C NMR, and MS analyses (experimental section). The IR spectra of compounds **4** and **5** did not show any absorption related to the nitrile function. The ^1^H NMR spectrum of **5** showed singlet at δ 6.44 ppm for two protons corresponding to amino group (–NH_2_). The aromatic protons were observed as multiplet at δ 7.42–7.57 ppm. The proton of thiadiazole ring resonates singlet at δ 8.51 ppm. The ^13^C NMR spectrum displayed ten carbon signals corresponding to twelve carbon atoms. The characteristic carbon signal of conjugated cyclic carbonyl group was recorded at δ 164.38 ppm.

**Figure 3:**
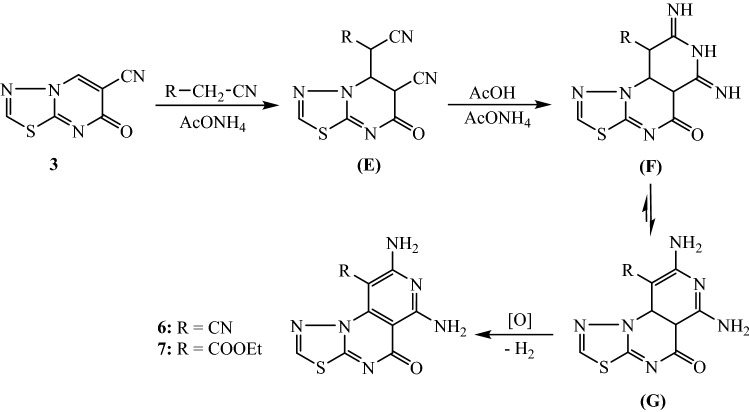
Preparation of 6,8-diaminopyridothiadiazolo[3,2-*a*]pyrimidine derivatives **6** and **7**.

The tricyclic 6,8-diaminopyridothiadiazolo[3,2-*a*]pyrimidines **6** and **7** were obtained by the treatment of thiadiazolo[3,2-*a*]pyrimidine derivative **3** with active nitrile components (namely, malononitrile and ethyl cyanoacetate). The reaction was conducted by heating the reactants in acetic acid and ammonium acetate (Fig. 3). The structures of **6** and **7** were elucidated from the results of the spectral analyses. The proposed mechanism for the reaction of thiadiazolo[3,2-*a*]pyrimidine compound **3** with activated nitrile involves the nucleophilic addition of nitrile through its methylene group to the cyclic unsaturated nitrile of compound **3** to yield the intermediate Michael adduct **(E)**. The heterocyclization of the intermediate **(E)** was assumed to occur by the addition of ammonia to the nitrile groups to produce the imino-perhydropyridine intermediate **(F)**. The tautomerization leading to the aminodihydropyridine intermediate **(G)**, followed by air oxidation (loss of H_2_) results in the formation of the pyridothiadiazolo[3,2-*a*]pyrimidine compounds **6** and** 7** (Fig. 3).

**Figure 4:**
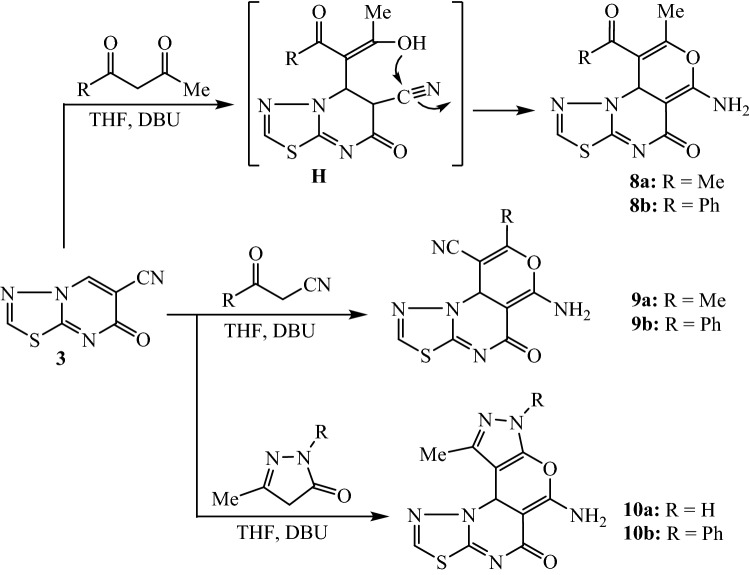
Synthesis of pyranothiadiazolo[3,2-*a*]pyrimidine derivatives **8**, **9** and **10**.

The pyrano[3,4-*e*]thiadiazolo[3,2-*a*]pyrimidine tricyclic and tetracyclic compounds **8**, **9** and **10** were obtained by the reaction of thiadiazolo[3,2-*a*]pyrimidine derivative **3** with acetylacetone and benzoyl acetone as examples from diketones, acetyl acetonitrile and benzoyl acetonitrile as examples from ketonitriles and 3-methylpyrazolin-5-ones, respectively (Fig. 4). The reaction was carried out by heating the reactants in tetrahydrofuran, which was initiated by using the 1,8-diazabicyclo[5.4.0]undec-7-ene (DBU) protocol. The suggested mechanism involves the Michael addition of the enolate carbonyl reagent to the β-carbon of the unsaturated nitrile system. The produced intermediate **H** undergoes intramolecular cyclization by the addition of enolic-OH functionality to the nitrile group in order to yield the pyranothiadiazolo[3,2-*a*]pyrimidine ring systems **8a** and **8b**. The structures of compounds **8**, **9** and **10** were confirmed by the IR, ^1^H NMR, ^13^C NMR, and MS analyses. Accordingly, the ^1^H NMR spectrum of **8a** (as a typical example) exhibited two singlet signals at δ 2.18 and 2.34 ppm to identify the protons of methyl and acetyl groups, respectively. The proton of pyran ring is recorded at δ 5.26 ppm as singlet signal. The protons of amino function (-NH_2_) resonate as singlet at δ 6.79 ppm. The singlet at δ 8.28 was attributed to the proton of thiadiazole ring. The ^13^C NMR spectrum displayed eleven carbon signals. The characteristic carbon signal of acetyl-carbonyl carbon is observed at δ 195.91 ppm. The mass analysis recorded a molecular ion peak at m/z = 278.

**Figure 5:**
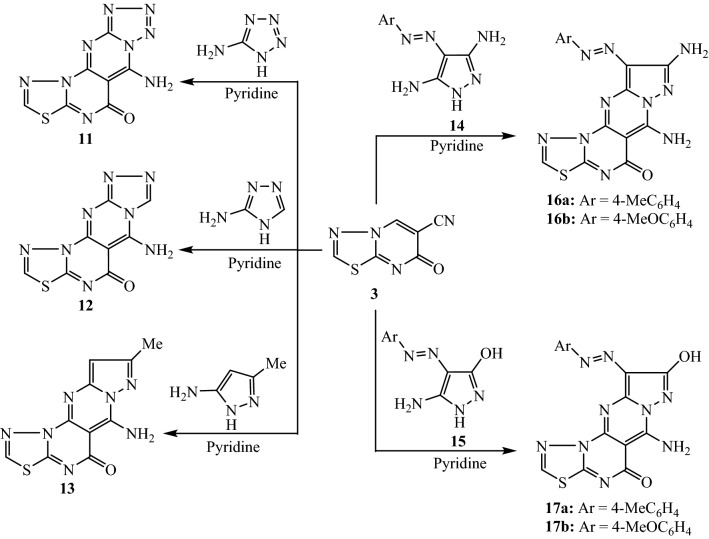
Reaction of thiadiazolopyrimidine derivative **3** with 3-aminotriazole, 5-aminotetrazole, and/or 5-aminopyrazoles.

The construction of pyrimidine nucleus fused with the building unit **3** has been explored through the reactions with various cyclic nitrogen 1,3-binucleophiles. Thus, the tetracyclic compounds **11**, **12**, and **13** were produced by the reaction of thiadiazolo[3,2-*a*]pyrimidine derivative **3** with different α-aminoazole reagents (namely, 5-aminotetrazole, 3-amino-1,2,4-triazole, and/or 5-amino-3-methylpyrazole) as 1,3-binucleophiles (Fig. 5). Finally, the tetracyclic derivatives, 6-amino-10-arylazo-pyrazolo[1′,5′:1,2]pyrimido[5,4-*e*]thiadiazolo[3,2-*a*]pyrimidin-5-ones **16** and **17**, were produced by the treatment of thiadiazolo[3,2-*a*]pyrimidine derivative **3** with 3,5-diamino-4-arylazopyrazole **14** and/or 5-amino-4-arylazopyrazol-3-ol **15**, respectively (Fig. 5). In general, the formation of the tetracyclic compounds **11**, **12**, **13**, **16,** and **17** involves the heating of compound **3** with the aminoazole reagent in pyridine for four hours. The chemical structures were characterized from the mutually consistent data obtained from the spectral analyses (IR, ^1^H NMR, ^13^C NMR, and MS).

### Biological assessment

#### Antimicrobial and antiquorum-sensing assessment

The antimicrobial activity of the synthesized thiadiazolopyrimidine compounds was studied toward diverse pathogenic strains, such as gram-positive bacteria (*Staphylococcus aureus* ATCC 29213 and *Bacillus cereus* UW85), gram-negative bacteria (*Escherichia coli* ATCC 12435 and *Pseudomonas aeruginosa* ATCC 29260), and fungi (*Candida albicans* and *Aspergillus fumigatus* 293). The assessment was performed through the two-fold dilution technique using Ampicillin (antibacterial) and Fluconazole (antifungal) as the reference drugs^28,29^. The minimal inhibitory concentration (MICs, µg/mL) of the synthesized derivatives for prohibiting microbial growth was determined through the visual detection (no turbidity) technique. The obtained results (Table 1) for the four bacterial strains indicated that the compounds **8a–b** and **9a–b** demonstrated significant efficacy toward the two strains of gram-positive bacteria (*S. aureus* and *B. cereus*). Meanwhile, derivatives **16a–b** and **17a–b** revealed eminent effectiveness toward gram-negative bacteria, such as *E. coli* and *P. aeruginosa*. Furthermore, compounds **8a–b** and **9a–b** showed good effectiveness toward *C. albicans* but no marked activity against *A. fumigatus* (Table 1).

**Table 1:** Antibacterial and antifungal efficacy for the synthesized thiadiazolopyrimidine compounds.

Cpd. no.	MIC (µg/mL)					
	Gram +ve bacteria		Gram −ve bacteria		Fungi	
	*S. aureus*	*B. cereus*	*E. coli*	*P. aeruginosa*	*C. albicans*	*A. fumigatus*
**3**	1250	> 1250	> 1250	> 1250	> 1250	> 1250
**4**	1250	> 1250	> 1250	> 1250	> 1250	> 1250
**5**	625	625	1250	1250	1250	> 1250
**6**	> 1250	1250	> 1250	1250	> 1250	> 1250
**7**	1250	1250	625	1250	> 1250	> 1250
**8a**	312.5	625	1250	1250	312.5	> 1250
**8b**	156	312.5	1250	312.5	156	1250
**9a**	312.5	312.5	625	625	312.5	> 1250
**9b**	312.5	156	625	625	312.5	> 1250
**10a**	625	625	1250	312.5	625	> 1250
**10b**	625	625	312.5	625	> 1250	> 1250
**11**	312.5	1250	> 1250	1250	1250	> 1250
**12**	625	> 1250	> 1250	> 1250	> 1250	> 1250
**13**	1250	> 1250	1250	> 1250	1250	> 1250
**16a**	1250	> 1250	78	156	> 1250	> 1250
**16b**	625	> 1250	19.5	78	> 1250	> 1250
**17a**	625	625	156	156	> 1250	1250
**17b**	1250	625	19.5	39	1250	1250
**Ampicillin**	> 1250	1250	19.5	78	–	–
**Fluconazole**	–	–	–	–	> 1250	> 1250

Ampicillin is the reference drug of bacteria; Fluconazole is the reference of fungi.

The synthesized thiadiazolopyrimidine-based compounds were screened for their antiquorum-sensing (anti-QS) inhibition activity by the *Chromobacterium violaceum* (ATCC 12472) technique using catechin as the standard compound^28–30^. The QS technique of *C. violaceum* was remarked by the detection of violacein (violet pigment)^31,32^. Meanwhile, the reactivity of the synthesized thiadiazolopyrimidine-containing derivatives as drugs depends on their efficiency to inhibit the liberation of violacein during the QS technique. The QS inhibition was determined by the following equation: QS inhibition (mm) = (r_2_ − r_1_), where r_1_ is the inhibition radius of the bacterial growth and r_2_ is the inhibition radius for growth as well as the release of the pigment. The compounds **8b**, **9a**, **16a–b,** and **17a–b** exhibited remarkable anti-QS activities (Table 2).

**Table 2:** Quorum-sensing inhibitor efficacy of the synthesized thiadiazolopyrimidine derivatives.

Cpd. no.	Diameter of quorum-sensing inhibition (mm)^a^ *C. violaceum*
**3**	–
**4**	–
**5**	–
**6**	–
**7**	–
**8a**	3
**8b**	12
**9a**	11
**9b**	3
**10a**	13
**10b**	9
**11**	5
**12**	3
**13**	5
**16a**	9
**16b**	12
**17a**	11
**17b**	12
Catechin	2

^a^No effectiveness (–, < 2 mm inhibition zone); weak effectiveness (2–9 mm); moderate effectiveness (10–15 mm); strong effectiveness (> 15 mm).

*Structural activity relationship.* The relationship between the structures of the synthesized thiadiazolopyrimidine-containing compounds and achieved antimicrobial results was discussed as follow: (1) The incorporation of a pyrazole ring to the thiadiazolopyrimidine skeleton to produce 3-aminopyrazolo-thiadiazolopyrimidinone and 3-amino-phenylpyrazolothiadiazolo-pyrimidinone (compounds **4** and **5**) did not boost the activity toward all the screened bacterial strains. (2) The introduction of a pyran ring to the thiadiazolopyrimidine skeleton in compounds **8a–b**, **9a–b** and **10a–b** promoted promising antibacterial effectiveness against *S. aureus* and *B. cereus*. In addition, they demonstrated remarkable antifungal efficacy toward *C. albicans* and no significant activity against *A. fumigatus*. (3) The tetracyclic pyrazolopyrimido-thiadiazolopyrimidinone derivatives **16a–b** and **17a–b** displayed good results against *E. coli* and *P. aeruginosa*, which may be attributed to the existence of amino and hydroxyl groups at the six and nine positions. (4) The replacement of the methyl group in compound **9a** by the phenyl ring caused favorable effectiveness against *B. cereus* (derivative **9b**), while the replacement of the nitrile group on the pyran ring (derivative **9a**) by the acetyl group (derivative **8a**) led to eminent antimicrobial effectiveness toward gram-positive as well as gram-negative bacteria. (5) On the other hand, the incorporation of tetrazole and/or triazole moieties to the thiadiazolopyrimidine skeleton (compounds **11** and **12**) diminished the activity toward all the examined microbes.

#### In vitro cytotoxicity evaluation

The cytotoxicity of the prepared tri-and tetra-cyclic thiadiazolopyrimidine compounds were examined toward various cancer cell lines, such as liver and breast cancer (MCF-7), prostate cancer (PC3), laryngeal carcinoma (Hep-2), carcinoma (HepG2), and standard normal fibroblast cells (WI38) using the MTT assay^33^ at the National Research Centre (Egypt). The cytotoxicity (Table 3) toward 50% inhibition of the cell viability (IC_50_ values) was assessed using 5-fluorouracil (5-Fu) as the standard anticancer drug. The thiadiazolopyrimidine derivatives **16a, 16b**, **17a,** and **17b** exhibited the highest cytotoxic efficacy against the MCF-7 cell lines with the IC_50_ values of 7.53, 5.69, 9.36, and 8.84 µM, respectively. The thiadiazolopyrimidine derivatives **8b** and **9b** presented strong efficacy against the MCF-7 and Hep-2 cell lines, with the IC_50_ values ranging from 11.71 to 14.68 µM. Also, these compounds displayed moderate activity against the HepG2 and PC3 cell lines, as observed from their IC_50_ values ranging from 21.62 to 38.36 µM. The pyrazolo-thiadiazolopyrimidinone compounds **4** and **5** showed strong activity against the HepG2 cells with the IC_50_ values of 15.36 and 19.16 µM, respectively. Furthermore, compounds **16** and **17** showed moderate effectiveness against the three cell lines by following the order: Hep-2 ˃ HepG2 ˃ PC3.

**Table 3:** Cytotoxic activities of the synthesized derivatives.

Cpd. no.	Cytotoxicity IC_50_ (μM)				
	HepG2	Hep-2	PC3	MCF-7	WI38
**3**	82.33 ± 2.19	87.51 ± 2.35	92.81 ± 3.48	77.95 ± 1.37	07.82 ± 1.33
**4**	15.36 ± 0.45	78.36 ± 2.20	72.31 ± 2.18	61.21 ± 1.11	09.35 ± 0.21
**5**	19.16 ± 0.52	61.25 ± 1.51	61.83 ± 1.68	54.75 ± 1.48	10.91 ± 0.83
**6**	62.24 ± 1.07	57.65 ± 1.72	66.26 ± 2.49	49.56 ± 1.25	14.11 ± 0.36
**7**	36.73 ± 0.17	34.19 ± 0.93	61.83 ± 1.35	39.82 ± 0.68	43.78 ± 0.52
**8a**	37.12 ± 0.23	22.61 ± 0.21	34.44 ± 0.64	33.42 ± 0.23	27.49 ± 0.60
**8b**	21.62 ± 0.14	14.65 ± 0.43	30.64 ± 0.57	14.68 ± 0.16	42.82 ± 1.06
**9a**	33.17 ± 0.36	19.98 ± 0.24	46.43 ± 1.07	22.66 ± 0.29	36.12 ± 0.28
**9b**	26.78 ± 0.18	11.71 ± 0.36	38.36 ± 0.89	12.84 ± 0.26	48.16 ± 1.47
**10a**	41.98 ± 1.24	34.04 ± 0.46	40.11 ± 0.36	24.21 ± 0.52	23.26 ± 0.60
**10b**	37.59 ± 1.26	28.76 ± 1.80	27.26 ± 0.53	27.44 ± 0.49	25.41 ± 0.62
**11**	42.18 ± 1.17	38.76 ± 0.74	38.10 ± 0.49	31.81 ± 0.67	20.24 ± 0.39
**12**	44.28 ± 1.35	34.15 ± 0.97	47.38 ± 0.89	36.92 ± 0.29	17.66 ± 0.71
**13**	28.63 ± 0.41	30.16 ± 0.59	35.56 ± 0.88	57.63 ± 1.40	44.36 ± 1.24
**16a**	37.59 ± 1.26	32.74 ± 0.36	44.18 ± 0.94	07.53 ± 0.08	60.07 ± 2.41
**16b**	42.34 ± 0.75	38.25 ± 0.57	47.92 ± 1.48	05.69 ± 0.39	62.26 ± 3.03
**17a**	38.27 ± 0.17	29.58 ± 0.15	39.40 ± 2.01	09.36 ± 0.23	57.86 ± 1.18
**17b**	33.54 ± 0.25	18.97 ± 0.40	36.75 ± 0.43	08.84 ± 0.17	58.37 ± 2.29
**5-Fu**^**a**^	07.20 ± 0.45	05.35 ± 0.23	08.78 ± 0.60	05.58 ± 0.31	10.32 ± 0.62

^a^5-Fluorouracil (5-Fu) is the reference drug for anticancer tests.

The suggested structural activity relationship (SAR) of the thiadiazolopyrimidine derivatives suggested the structural countenance associated with the anticancer efficacy. (1) The pyrazole ring fused with the thiadiazolopyrimidinone skeleton in compounds **4** and **5** caused strong activity against the HepG2 cells and low effectiveness against the other three tested cells. (2) The incorporation of pyridine to the thiadiazolopyrimidine skeleton in compounds **6** and **7** did not offer the desired activity against the tested cell lines. In contrast, the fusion of the pyran ring to the thiadiazolopyrimidine skeleton in compounds **8b** (substituted with benzoyl group) and **9b** (substituted with phenyl group) presented strong anticancer efficacy against the MCF-7 and Hep-2 cells and reasonable activity against the HepG2 and PC3 cell lines. (3) The construction of the pyrazolopyrimidine moiety fused with the thiadiazolopyrimidine skeleton to produce tetracyclic compounds led to the enhancement of the anticancer activity against the MCF-7 cell lines. In addition, compounds **16a** and **16b** possessing an aminopyrazole nucleus exhibited higher cytotoxic efficacy against the MCF-7 cell lines than their corresponding compounds **17a** and **17b** containing a hydroxypyrazole nucleus. Also, the derivatives **16b** and **17b** (substituted with the 4-anisyl group) displayed higher reactivity than their conjugates **16a** and **17a** containing the 4-tolyl group. This is supported by order of biological anticancer activity toward MCF-7 cell lines on tuning the substituents^34^. (4) The results of the cytotoxicity examination on normal cells (WI38) indicated that compounds **16** and **17** displayed the lowest cytotoxicity with the IC_50_ values ranging from 57.86 to 62.26 µM. (5) The tetracyclic compounds **16a** and **16b** showed promising selectivity as cytotoxic agents against the MCF-7 cells with weak cytotoxic effects on normal cells (WI38).

*Bleomycin-dependent DNA damage.* The prepared polycyclic thiadiazolopyrimidine-based compounds were examined through the bleomycin-dependant DNA damage, and the results were compared to that of ascorbic acid as a positive control. The obtained data reflected the ability of these derivatives to protect the DNA from damage. The capability of compounds **16a–b** and **17a–b** to manifest the best protective effect against DNA damage was indicated by the corresponding absorbance values ranging from 0.031 to 0.053 (Table 4)^35,36^.

**Table 4:** Bleomycin-dependent DNA damage of the synthesized thiadiazolopyrimidine scaffolds.

Cpd. no.	Absorbance^a^
**3**	0.281 ± 0.22
**4**	0.165 ± 0.19
**5**	0.146 ± 0.16
**6**	0.135 ± 0.14
**7**	0.132 ± 0.19
**8a**	0.078 ± 0.13
**8b**	0.066 ± 0.05
**9a**	0.064 ± 0.06
**9b**	0.083 ± 0.08
**10a**	0.098 ± 0.07
**10b**	0.119 ± 0.13
**11**	0.113 ± 0.16
**12**	0.107 ± 0.08
**13**	0.128 ± 0.11
**16a**	0.039 ± 0.07
**16b**	0.031 ± 0.04
**17a**	0.053 ± 0.05
**17b**	0.042 ± 0.03
**Ascorbic acid**	0.060 ± 0.02

^a^Values are mean for three replicates ± SD.

#### Molecular docking

The in silico molecular docking studies were conducted to evaluate the types of requisite interaction between the thiadiazolopyrimidine-based compounds and the crystal structure of the potent inhibitors of NUDT5 silence hormone signaling in breast cancer (PDB Code-5NQR)^37^. The thiadiazolopyrimidine derivative **3** displayed two types of intermolecular interactions with low binding effects. The first type of interaction bonds the S atom of the thiadiazole ring with Asp 194, and the second interaction bonds the N-atom in the nitrile group with Gly 61 over a binding score S of – 4.3922 kcal/mol (Fig. S1). The tricyclic compound **4** (pyrazolothiadiazolopyrimidine substituted with the amine functional group at position-3) showed two H-bonds resulting from the bonding of the N atom of pyrimidine with Arg 84 (2.92 Å) (Fig. S2) and that of the O atom of the carbonyl group with Arg 84. The resultant binding score S was found to be – 4.4293 kcal/mol. The pyrazolothiadiazolopyrimidine compound **5** exhibited a better binding score (S = – 4.2102 kcal/mol) through the formation of four H-bonds (Fig. S3). One of the H-bond resulted from the bonding of the S atom of the thiadiazole ring with Glu 169, the second bond formed between the N atom of the aminopyrazole moiety and Ser 172, while the third and fourth bonds were π–π interactions of the thiazole and pyrimidine rings with Ile 171.

Nonetheless, the pyridothiadiazolopyrimidine derivative **6** presented two H-bonds corresponding to the bonding of the N atom of aminopyridine with Arg 51 and that of the S atom of thiadiazole with Glu 65 (Fig. S4). The derivative **6** displayed weak interactions with the 5NQR amino acids (S = – 4.5643 kcal/mol). Meanwhile, the pyridothiadiazolopyrimidine compound **7** revealed two H-bonds between the N atom of the aminopyridine moiety with Asp 194, and the O atom of the ester group with Arg 51 of 5NQR (S = – 6.6614 kcal/mol) (Fig. S5). An H-bonding was formed by the N atom of the amidic moiety with Asp 347, and a π-π bond was observed between the pyridine ring and Arg 84 (3.40 Å).

**Figure 6:**
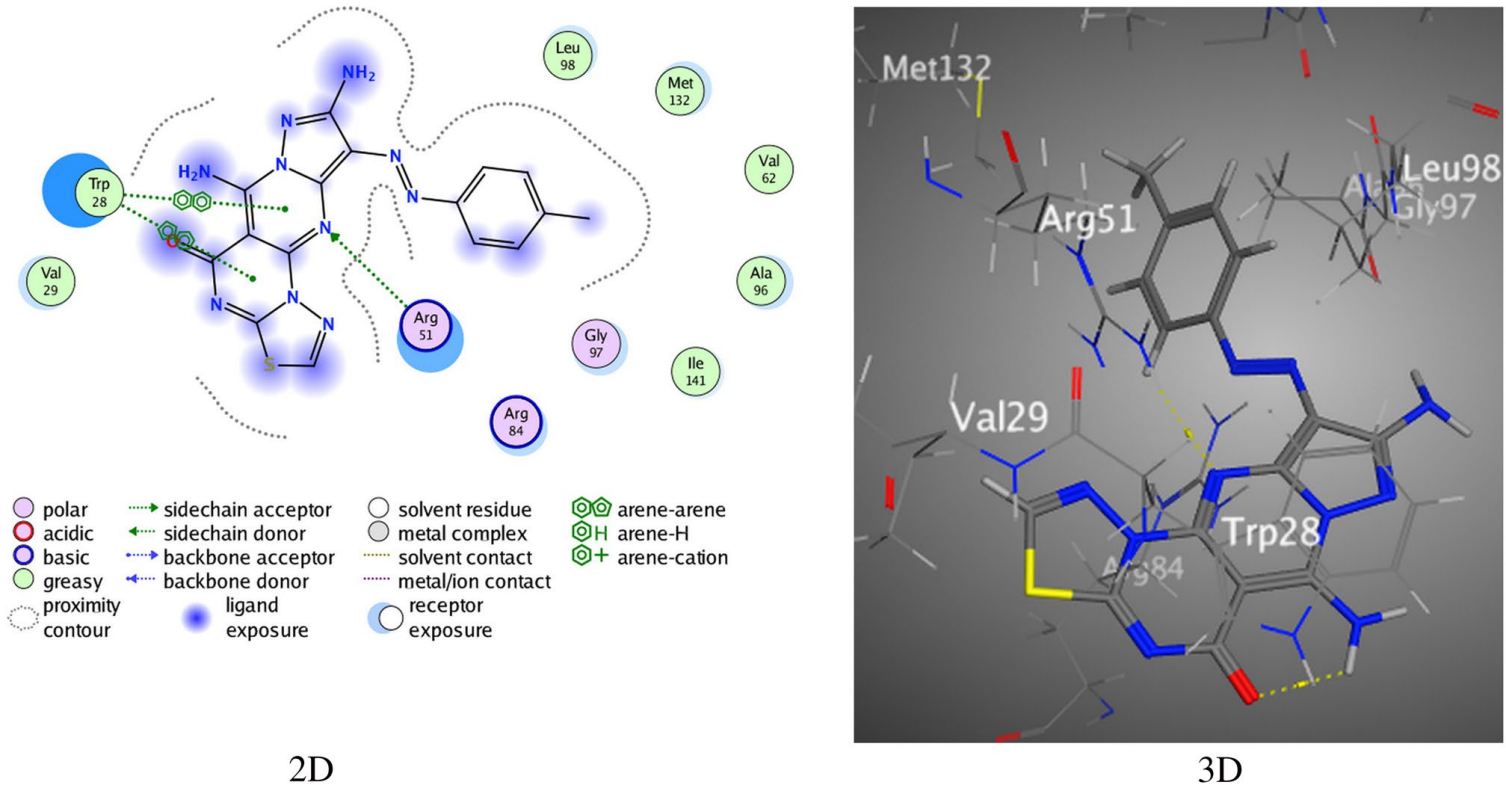
The interactions between **16a** and (PDB ID: 5NQR).

Similarly, the aminopyranothiadiazolopyrimidine derivative **8a** displayed two H-bonds resulting from the bonding of the S atom of thiadiazole ring with Asp 194 and N atom of the aminoxazine moiety with Cys 139 (S = – 5.7796 kcal/mol) (Fig. S6). While, compound **8b** showed three intermolecular forces resulting from the thiadiazole ring, one H-bond formed by the S atom of thiadiazole with Cys 139, and two π–π interactions with Arg 51 and Met 132 over a binding score S of – 5.9214 kcal/mol (Fig. S7). Also, derivative **9a** demonstrated two H-bonds resulting from the bonding of the S atom of thiadiazole with Asp 194 and N atom of the nitrile group with Gly along with a binding energy score, S of – 5.3557 kcal/mol (Fig. S8). Compound **9b** exhibited two H-bonds between the N atom of the amino group bonded to Ala 96 and Arg 84 through a good score S of – 6.1407 kcal/mol (Fig. S9). Moreover, the aminopyrazolopyrano-thiadiazolopyrimidines **10a** and **10b** exhibited H-bonds and π–π interactions. The derivative **10a** demonstrated three H-bonds between the N atom of the amino group and Val 62, N1 of pyrazole and Ala 96, and N2 of pyrazole and Arg 84 in addition to the π-π interaction between the thiadiazole ring and Arg 51. The bonds resulted in an overall energy score (S) of – 4.9886 kcal/mol (Fig. S10). Besides the three π-π interactions displayed by thiadiazole with Val 170, pyrazole with Ile 171, and phenyl with Ile 171, the derivative **10b** demonstrated two H-bonds formed by the S atom in thiadiazole with Thr 117, and N1 of the pyrazole ring with Ser 172. The binding score of **10b** was found to be – 5.2193 kcal/mol, as shown in Fig. S11. Moreover, the tetracyclic structures **11–13** revealed reasonable binding scores from − 5.2800 to – 5.4772 kcal/mol resulting from different hydrogen bonds and π-π interactions (Table 5, Figs. S12, S13, and S14). Furthermore, the aminopyrazolopyrimidothiadiazolopyrimidines **16** and **17** displayed remarkable binding scores. For instance, derivative **16a** exhibited a binding score of – 6.2989 kcal/mol (Fig. 6) attributed to the H-bonding of the N atom of pyrimidine with Arg 51, and the two π–π interactions of Ter 28 with pyrimidothiadiazole and pyrimidopyrimidine, respectively.

**Table 5:** The binding interaction of the synthesized thiadiazolopyrimidine derivatives.

Code	S (energy score) (Kcal/mol)	Rmsd (refine unit)	Interaction with ligand	Types of interactions	Distance (Å)
**3**	− 4.3922	1.0737	S of Thiadiazole ring with Asp 194	H-donor	3.13
			N of nitrile group with Gly 61	H-acceptor	3.70
**4**	− 4.4293	1.5133	N of pyrimidine ring with Arg 84	H-acceptor	3.47
			O of Carbonyl group with Arg 84	H-acceptor	3.23
**5**	− 4.7102	0.7663	S of Thiadiazole ring with Glu 169	H-donor	3.34
			N of amino pyrazole with Ser 172	H-donor	2.98
			Thiazole ring with Ile 171	π–π interaction	4.05
			Pyrimidine ring with Ile 171	π–π interaction	4.29
**6**	− 4.5643	1.0767	N of amino pyridine with Arg 51	H-donor	2.98
			S of Thiadiazole ring with Glu 65	H-donor	3.97
**7**	− 5.5334	1.3303	N of amino pyridine with Asp 194	H-donor	3.16
			O of ester group with Arg 51	H-acceptor	2.93
**8a**	− 5.4796	1.2247	S of Thiadiazole ring with Asp 194	H-donor	3.24
			N of amino Oxazine with Cys 139	H-donor	3.96
**8b**	− 5.7214	0.7835	S of Thiadiazole ring with Cys 139	H-donor	3.59
			Thiadiazole ring with Arg 51	π–π interaction	4.00
			Thiadiazole ring with Met 132	π–π interaction	4.22
**9a**	− 5.3557	0.9982	S of Thiadiazole ring with Asp 194	H-donor	3.06
			N of nitrile group with Gly 61	H-acceptor	3.64
**9b**	− 6.4407	1.1913	N of amino Oxazine with Ala 96	H-donor	2.82
			N of amino Oxazine with Arg 84	H-acceptor	3.09
**10a**	− 4.9886	0.9778	N of amino Oxazine with Val 62	H-donor	2.97
			N1 of Pyrazole ring with Ala 96	H-donor	3.04
			N2 of Pyrazole ring with Arg 84	H-acceptor	3.18
			Thiadiazole ring with Arg 51	π–π interaction	3.23
**10b**	− 5.2193	1.0414	S of Thiadiazole ring with Thr 117	H-donor	3.71
			NH of pyrazole ring with Ser 172	H-acceptor	3.17
			Thiadiazole ring with Val 170	π–π interaction	3.71
			pyrazole ring with Ile 171	π–π interaction	4.38
			Phenyl ring ring with Ile 171	π–π interaction	3.77
**11**	− 5.2800	1.1608	N of Amino group with Met 132	H-donor	3.95
			N of pyrimidine ring with Arg 84	H-acceptor	2.89
			Thiadiazole ring with Gly 97	π–π interaction	3.75
**12**	− 5.4282	1.2159	S of Thiadiazole ring with Glu 93	H-donor	3.18
			O of Carbonyl group with Arg 84	H-acceptor	2.91
**13**	− 5.4772	1.3030	N of amino Pyrimidine with Met 132	H-donor	4.01
			N of Thiadiazole ring with Arg 84	H-acceptor	3.19
			Thiadiazole ring with Gly 97	π–π interaction	3.79
			pyrimidine ring with Gly 97	π–π interaction	3.92
**16a**	− 6.2989	1.3113	N of pyrimidine ring with Arg 51	H-acceptor	3.33
			pyrimidothiazdiazolering with Ter 28	π–π interaction	3.60
			pyrimidopyrimidine ring with Ter 28	π-π interaction	3.67
**16b**	− 7.7053	0.4672	N of aminopyrazole with Ala 96	H-donor	3.09
			N of aminopyrazole with Gln 82	H-acceptor	3.28
**17a**	− 7.4560	0.5501	O of Hydroxyl group with Asp 194	H-donor	3.18
**17b**	− 7.5846	0.8285	N of aminopyrimidine with Asp 194	H-donor	3.01
			Phenyl ring with Arg 51	π–π interaction	4.68
**5-Fu**	− 3.8546	0.8432	N1 of pyrimidine ring with Cys139	H-donor	3.93

**Figure 7:**
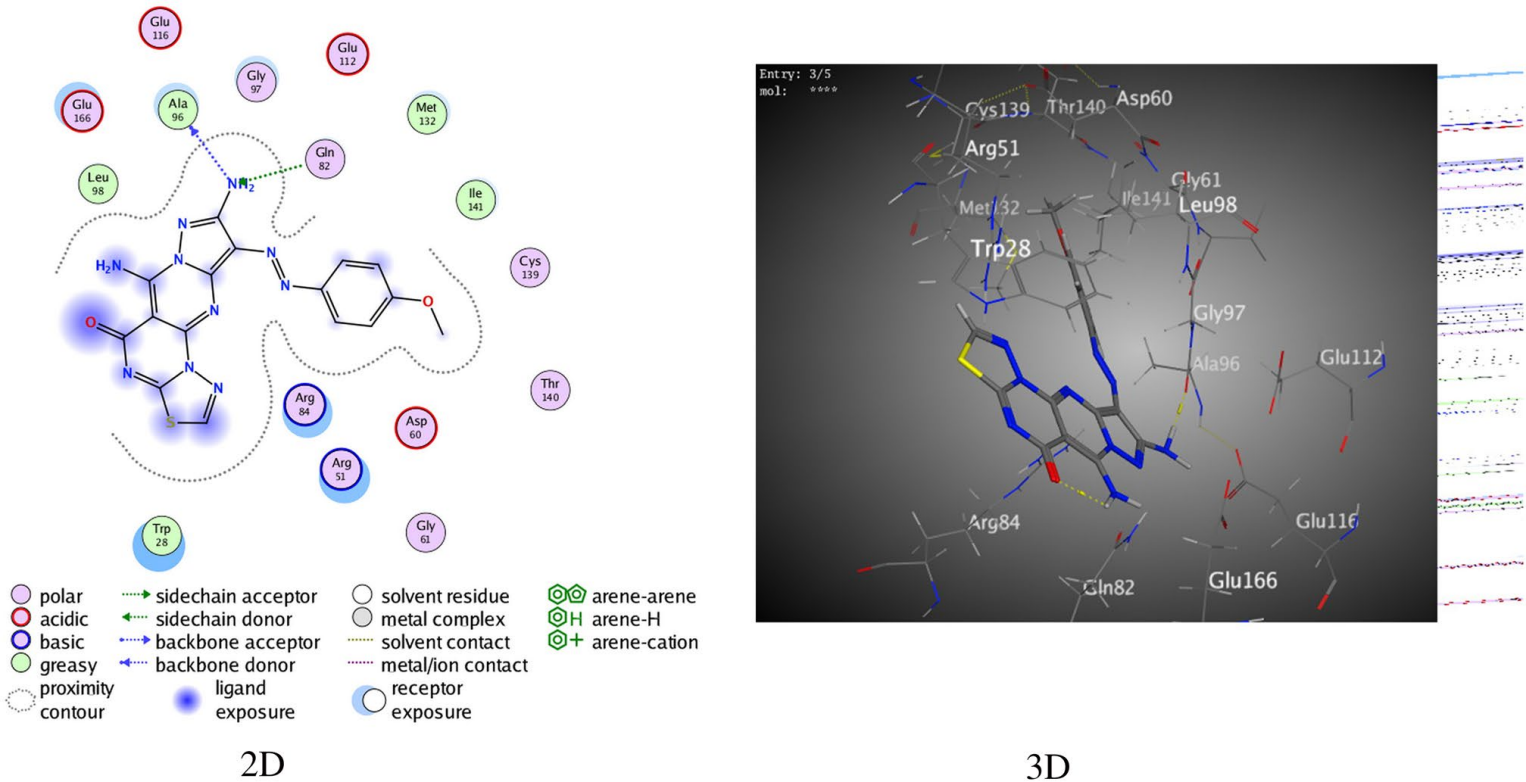
The interactions between **16b** and (PDB ID: 5NQR).

Compound **16b** exhibited two intermolecular hydrogen bonds resulting from the N atom of aminopyrazole with Ala 96 and Gln 82 through a binding score (S) of – 7.7053 kcal/mol (Fig. 7).

Alternatively, derivative **17a** showed a binding score of – 7.4560 kcal/mol (Fig. S15) resulting from the H-bond of the O atom in the hydroxyl group with Asp 194. Finally, derivative **17b** showed π–π binding between the phenyl ring and Arg 51, and an H-bond between the N atom of the aminopyrimidine moiety and Asp 194 over a binding score of – 7.5846 kcal/mol (Fig. S16). The standard reference drug 5-fluorouracil was subjected to 5NQR for a comparative study of the synthesized derivatives. The drug presented an intermolecular hydrogen bond with a binding score of – 7.4560 kcal/mol (Fig. S17).

Finally, the docking technique showed that the derivatives **16a**, **16b**, **17a**, and **17b** gave respectable binding scores of – 6.2989, – 7.7053, – 7.4560, and – 7.5846 kcal/mol, respectively, in contrast to 5-Fluorouracil exhibiting a binding score of – 3.8546 kcal/mol with 5NQR. The two-and three-dimensional images of most of the derivatives presented two intramolecular hydrogen bonds resulting from the thiadiazole and pyrimidine moieties. The challenge in the docking method is the development of the level of conformation for the ligand interactions in distinct compounds depending on the binding scores. All the synthesized derivatives possess thiadiazole and pyrimidine moieties that form hydrogen bonds with the receptors and chemically disparate amino acids of 5NQR. The large pocket size of 5NQR was constrained by typically few polar residues with specific binding sites (Asp 194, Ter 28, Met 132, Arg 84, Gly 97 and Cys139), as observed from the three-dimensional images, and offered a proper cavity for the synthesized thiadiazolopyrimidine-based compounds.

## Conclusion

Seventeen tricyclic and tetracyclic compounds containing the thiadiazolo[3,2-*a*]pyrimidine ring system were synthesized. The synthetic strategy for the preparation of the tricyclic compounds, pyrazolothiadiazolo-pyrimidines, pyridothiadiazolo-pyrimidines, and pyranothiadiazolo-pyrimidines, was based on the reactions of 6-cyano-7-oxo-7*H*-[1,3,4]thiadiazolo[3,2-*a*]pyrimidine **3** with various nitrogen and carbon-containing nucleophilic reagents. The tetracyclic ring systems formed from pyrimido[5,4-*e*]thiadiazolo[3,2-*a*]pyrimidine skeleton fused with various azoles, such as tetrazole, triazole and/or pyrazoles, were prepared by the cyclization of the building block **3** with different α-aminoazole reagents. The targeted thiadiazolopyrimidine compounds were evaluated for their antimicrobial efficacy against the two types of bacterial strains along with the antifungal strains. The results demonstrated that the derivatives **8a–b** and **9a–b** with the pyranothiadiazolopyrimidine nucleus displayed significant activities against *S. aureus* and *B. cereus*. Meanwhile, the derivatives **16a–b** and **17a–b** showed prominent efficiencies against *E. coli* and *P. aeruginosa*. The synthesized thiadiazolopyrimidine compounds were tested by antiquorum-sensing, where the derivatives **16a–b**, **17a–b**, **8b** and **9a** demonstrated acceptable activities. The relationship between the chemical structures and recognized antimicrobial results was determined. The in vitro antitumor efficiency of the synthesized thiadiazolopyrimidine scaffolds toward the four cancer cells (MCF-7, PC3, Hep-2, and HepG2) and lung normal cell (WI38) was examined by employing the MTT technique. The tetracyclic pyrazolopyrimido[5,4-*e*]thiadiazolo[3,2-*a*]pyrimidine derivatives **16a, 16b**, **17a** and **17b** recorded potent cytotoxic efficacy against the MCF-7 cancer cells. Also, the endorsed structural activity relationship of the synthesized thiadiazolopyrimidines provided a correlation between their chemical structures and anticancer efficiency. The in silico-docking studies (PDB Code-5NQR) indicated that the compounds **16b** and **17b** exhibited the highest binding scores (-7.7053 and –7.5846 kcal/mol).

## Experimental

### Chemistry

The IR spectra were recorded using the Thermo Scientific Nicolet iS10 FTIR spectrometer using KBr discs. The ^1^H NMR (500 MHz) and ^13^C NMR (125 MHz) spectra were obtained in DMSO-*d*_6_ using the JEOL’s NMR spectrometer. The mass analyses were performed at 70 eV on the Shimadzu Qp-2010 plus mass spectrometer. The elemental analyses were conducted using the EuroVector instrument analyzer (EA3000 Series).

#### Synthesis of 2-cyano-3-(dimethylamino)-N-(1,3,4-thiadiazol-2-yl)acrylamide (2)

In a 100 ml round bottom flask (RBF), 2-(cyanoacetamido)-1,3,4-thiadiazole **(1)** (1.68 g, 10 mmol) dissolved in 25 ml of dioxane was treated with *N*–*N*-dimethylformamidedimethylacetal (1.20 ml, 10 mmol). The mixture was heated for 4 h after, which the product was formed as an orange solid that was collected and dried.

Yield 71%, m.p. = 226–227 °C, lit. m.p. = 224 °C^27^. IR: 3221 (N–H), 2196 (C≡N), 1678 cm^−1^ (C=O). ^1^H NMR: δ 3.13 (s, 3H), 3.23 (s, 3H), 7.91 (s, 1H, CH = C), 9.04 (s, 1H, thiadiazole-H), 10.26 ppm (s, 1H). Anal. Calcd. for C_8_H_9_N_5_OS (223.05): C, 43.04; H, 4.06; N, 31.37%. Found: C, 43.13; H, 4.01; N, 31.43%.

#### Synthesis of 6-cyano-7-oxo-7H-[1,3,4]thiadiazolo[3,2-a]pyrimidine (3)

A solution of thiadiazolyl-acrylamide compound **2** (2.23 g, 10 mmol) in 25 ml glacial acetic acid was refluxed for 4 h. The obtained solid was diluted with 50 mL of cold water, which was collected and purified by recrystallization in dioxane.

Yield 56%, m.p. = 264–265 °C. IR: 2229 (C≡N), 1690 cm^−1^ (C=O). ^1^H NMR: δ 8.53 (s, 1H, thiadiazole-H), 9.37 ppm (s, 1H, pyrimidine-H). ^13^C NMR: 94.84, 115.52, 142.30, 159.06, 161.29, 165.56 ppm. MS: m/z (%) = 178 (M^+^, 83.46). Anal. Calcd. for C_6_H_2_N_4_OS (178.00): C, 40.45; H, 1.13; N, 31.45%. Found: C, 40.31; H, 1.09; N, 31.50%.

#### Synthesis of 3-aminopyrazolo[4,3-e][1,3,4]thiadiazolo[3,2-a]pyrimidin-4-ones 4 and 5

In a 50 ml RBF, hydrazine hydrate or phenylhydrazine (3 mmol) was added to a solution of the thiadiazolopyrimidine compound **3** (0.53 g, 3 mmol) in a 3:1 mixture of EtOH and DMF. The above mixture was refluxed for 4 h. The resultant solid was collected and recrystallized in an EtOH/DMF mixture (1:1) to furnish the conforming pyrazolo[4,3-*e*]thiadiazolo [3,2-*a*]pyrimidine compounds **4** and **5**.

#### 3-Aminopyrazolo[4,3-e][1,3,4]thiadiazolo[3,2-a]pyrimidin-4(1H)-one (4)

Yield 68%, m.p. = 281–282 °C. IR: 3381, 3275, 3211 (NH_2_ and NH), 1664 cm^−1^ (C=O). ^1^H NMR: δ 6.58 (s, 2H), 8.36 (s, 1H, thiadiazole-H), 11.80 ppm (s, 1H). ^13^C NMR: 97.34, 144.61, 150.10, 152.08, 161.71, 164.25 ppm. MS: m/z (%) = 208 (M^+^, 56.41). Anal. Calcd. for C_6_H_4_N_6_OS (208.02): C, 34.61; H, 1.94; N, 40.37%. Found: C, 34.44; H, 2.01; N, 40.25%.

#### 3-Amino-1-phenylpyrazolo[4,3-e][1,3,4]thiadiazolo[3,2-a]pyrimidin-4(1H)-one (5)

Yield 75%, m.p. = 254–255 °C. IR: 3370, 3218 (NH_2_), 1661 cm^−1^ (C=O). ^1^H NMR: δ 6.44 (s, 2H), 7.42–7.57 (m, 5H), 8.51 ppm (s, 1H, thiadiazole-H). ^13^C NMR: 95.73, 123.50 (2C), 127.29, 129.68 (2C), 138.95, 144.33, 149.70, 152.69, 160.22, 164.38 ppm. MS: m/z (%) = 284 (M^+^, 44.93). Anal. Calcd. for C_12_H_8_N_6_OS (284.05): C, 50.70; H, 2.84; N, 29.56%. Found: C, 50.59; H, 2.80; N, 29.48%.

#### Synthesis of 6,8-diamino-5-oxo-pyrido[3,4-e][1,3,4]thiadiazolo[3,2-a]pyrimidines, 6 and 7

The thiadiazolopyrimidine compound **3** (0.89 g, 5 mmol) and malononitrile (or ethyl cyanoacetate) (0.005 mol) were taken in a 50 ml RBF. Further, ammonium acetate (1.00 g) and 20 mL of acetic acid were added to the reaction mixture, which was refluxed for 8 h. The mixture was diluted with 40 ml cold water, and the generated solid was collected and purified by recrystallization in acetic acid.

#### 6,8-Diamino-9-cyano-5-oxo-5H-pyrido[3,4-e][1,3,4]thiadiazolo[3,2-a]pyrimidine (6)

Yield 66%, m.p. = 302–303 °C. IR: 3381, 3304, 3248 (NH_2_ and N–H), 2211 (C≡N), 1667 cm^−1^ (C=O). ^1^H NMR: δ 7.14 (s, 1H), 8.27 (s, 1H), 8.45 (s, 1H, thiadiazole-H), 8.81 ppm (s, 2H). ^13^C NMR: δ 71.54, 90.33, 114.16, 138.08, 158.69, 162.84, 163.90, 166.21, 167.50 ppm. MS: m/z (%) = 259 (M^+^, 70.11). Anal. Calcd. for C_9_H_5_N_7_OS (259.03): C, 41.70; H, 1.94; N, 37.82%. Found: C, 41.86; H, 1.88; N, 37.72%.

#### Ethyl 6,8-diamino-5-oxo-5H-pyrido[3,4-e][1,3,4]thiadiazolo[3,2-a]pyrimidine-9-carboxylate (7)

Yield 61%, m.p. = 278–280 °C. IR: 3367, 3293, 3241 (NH_2_), 1689 (C=O), 1665 cm^−1^ (C=O). ^1^H NMR: δ 1.28 (t, *J* = 7.00 Hz, 3H), 4.28 (q, *J* = 7.00 Hz, 2H), 7.23 (s, 1H), 8.15 (s, 1H), 8.47 (s, 1H, thiadiazole-H), 8.72 ppm (s, 2H). ^13^C NMR: δ 14.30, 61.14, 83.92, 89.68, 139.27, 154.57, 162.30, 163.74, 165.05, 167.58, 168.46 ppm. MS: m/z (%) = 306 (M^+^, 33.27). Anal. Calcd. for C_11_H_10_N_6_O_3_S (306.05): C, 43.13; H, 3.29; N, 27.44%. Found: C, 43.01; H, 3.33; N, 27.46%.

#### Synthesis of 6-aminopyrano[3,4-e][1,3,4]thiadiazolo[3,2-a]pyrimidin-5-ones, 8 and 9

The thiadiazolopyrimidine compound **3** (0.89 g, 5 mmol) was dissolved in 15 ml THF taken in a 50 mL RBF. To this solution, the appropriate active methylene compound (namely, acetylacetone, benzoyl acetone, acetyl acetonitrile, and benzoyl acetonitrile) (0.005 mol) and DBU (0.45 mL, 3 mol%) were added. The mixture was refluxed for 4 h and then allowed to cool up to 25 °C. The solid was filtered and recrystallized using THF to form the conforming pyranothiadiazolopyrimidines **8** and **9**.

#### 9-Acetyl-6-amino-8-methyl-5H,9aH-pyrano[3,4-e][1,3,4]thiadiazolo[3,2-a]pyrimidin-5-one (8a)

Yield 72%, m.p. = 226–227 °C. IR: 3371, 3288, 3236 (NH_2_ and N–H), 1670 (C=O), 1658 cm^−1^ (C=O). ^1^H NMR: δ 2.18 (s, 3H), 2.34 (s, 3H), 5.26 (s, 1H, pyran-H), 6.79 (s, 2H), 8.28 ppm (s, 1H, thiadiazole-H). ^13^C NMR: δ 16.84, 27.50, 42.11, 92.09, 108.02, 138.67, 157.33, 162.46, 164.96, 166.58, 195.91 ppm. MS: m/z (%) = 278 (M^+^, 80.64). Anal. Calcd. for C_11_H_10_N_4_O_3_S (278.05): C, 47.48; H, 3.62; N, 20.13%. Found: C, 47.30; H, 3.70; N, 20.26%.

#### 6-Amino-9-benzoyl-8-methyl-5H,9aH-pyrano[3,4-e][1,3,4]thiadiazolo[3,2-a]pyrimidin-5-one (8b)

Yield 76%, m.p. = 239–241 °C. IR: 3346, 3294, 3260 (NH_2_ and N–H), 1667 cm^−1^ (C=O). ^1^H NMR: δ 2.31 (s, 3H), 5.08 (s, 1H, pyran-H), 6.76 (s, 2H), 7.46–7.73 (m, 5H), 8.23 ppm (s, 1H, thiadiazole-H). ^13^C NMR: δ 16.87, 44.29, 92.28, 105.33, 128.42 (2C), 129.51 (2C), 133.77, 138.15, 139.42, 155.04, 162.71, 165.08, 166.86, 192.60 ppm. MS: m/z (%) = 340 (M^+^, 52.83). Anal. Calcd. for C_16_H_12_N_4_O_3_S (340.06): C, 56.46; H, 3.55; N, 16.46%. Found: C, 56.31; H, 3.50; N, 16.37%.

#### 6-Amino-9-cyano-8-methyl-5-oxo-5H,9aH-pyrano[3,4-e][1,3,4]thiadiazolo[3,2-a]pyrimidine (9a)

Yield 68%, m.p. = 264–265 °C. IR: 3360, 3292, 3181 (NH_2_ and N–H), 2214 (C≡N), 1660 cm^−1^ (C=O). ^1^H NMR: δ 2.14 (s, 3H), 5.16 (s, 1H, pyran-H), 6.71 (s, 2H), 8.26 ppm (s, 1H, thiadiazole-H). ^13^C NMR: δ 17.16, 41.51, 79.06, 91.86, 117.63, 138.74, 161.24, 162.89, 165.18, 166.47 ppm. MS: m/z (%) = 261 (M^+^, 45.09). Anal. Calcd. for C_10_H_7_N_5_O_2_S (261.03): C, 45.97; H, 2.70; N, 26.81%. Found: C, 46.10; H, 2.63; N, 26.70%.

#### 6-Amino-9-cyano-5-oxo-8-phenyl-5H,9aH-pyrano[3,4-e][1,3,4]thiadiazolo[3,2-a]pyrimidine (9b)

Yield 62%, m.p. = 258–260 °C. IR: 3356, 3270, 3184 (NH_2_ and N–H), 2210 (C≡N), 1655 cm^−1^ (C=O). ^1^H NMR: δ 5.09 (s, 1H, pyran-H), 6.69 (s, 2H), 7.49–7.56 (m, 5H), 8.18 ppm (s, 1H, thiadiazole-H). ^13^C NMR: δ 40.94, 81.22, 92.19, 118.01, 127.66 (2C), 128.58 (2C), 129.21, 131.40, 138.36, 161.75, 162.50, 165.30, 166.57 ppm. MS: m/z (%) = 323 (M^+^, 58.35). Anal. Calcd. for C_15_H_9_N_5_O_2_S (323.05): C, 55.72; H, 2.81; N, 21.66%. Found: C, 55.93; H, 2.88; N, 21.90%.

#### Synthesis of 6-amino-10-methylpyrazolo[4′,3′:5,6]pyrano[3,4-e][1,3,4]thiadiazolo[3,2-a]pyrimidin-5-ones, 10a and 10b

In a 50 ml RBF, each of the 3-methylpyrazolone compounds (5 mmol) was mixed with a solution of the thiadiazolopyrimidine derivative **3** (0.89 g, 5 mmol in 15 ml THF) containing DBU (0.45 ml, 3 mol%). The mixture was refluxed for 4 h and cooled down to room temperature. The resultant solid was collected, dried, and purified by recrystallization in an EtOH/DMF mixture (1:1) to furnish the cyclic compounds **10a** and **10b**, respectively.

#### 6-Amino-10-methyl-8,10b-dihydro-5H-pyrazolo[4′,3′:5,6]pyrano[3,4-e][1,3,4]thiadiazolo[3,2-a]pyrimidin-5-one (10a)

Yield 64%, m.p. = 310–312 °C. IR: 3369, 3281, 2188 (NH_2_ and N–H), 1652 cm^−1^ (C=O). ^1^H NMR: δ 2.21 (s, 3H), 4.78 (s, 1H, pyran-H), 6.92 (s, 2H), 8.34 (s, 1H, thiadiazole-H), 12.31 ppm (s, 1H). ^13^C NMR: δ 13.22, 45.69, 95.78, 112.19, 138.93, 140.55, 160.73, 161.48, 163.08, 165.25 ppm. MS: m/z (%) = 276 (M^+^, 30.44). Anal. Calcd. for C_10_H_8_N_6_O_2_S (276.04): C, 43.47; H, 2.92; N, 30.42%. Found: C, 43.29; H, 2.81; N, 30.55%.

#### 6-Amino-10-methyl-8-phenyl-8,10b-dihydro-5H-pyrazolo[4′,3′:5,6]pyrano[3,4-e][1,3,4]thiadiazolo[3,2-a]pyrimidin-5-one (10b)

Yield 58%, m.p. = 266–267 °C. IR: 3344, 3250, 3204 (NH_2_ and N–H), 1646 cm^−1^ (C=O). ^1^H NMR: δ 2.23 (s, 3H), 4.73 (s, 1H, pyran-H), 7.08 (s, 2H), 7.47–7.75 (m, 5H), 8.30 ppm (s, 1H, thiadiazole-H). ^13^C NMR: δ 13.31, 45.85, 95.61, 113.96, 122.16 (2C), 126.47, 129.28 (2C), 137.83, 138.78, 144.18, 154.29, 160.44, 162.53, 165.70 ppm. MS: m/z (%) = 352 (M^+^, 39.14). Anal. Calcd. for C_16_H_12_N_6_O_2_S (352.07): C, 54.54; H, 3.43; N, 23.85%. Found: C, 54.68; H, 3.50; N, 23.76%.

### Synthesis of tetracyclic compounds 11, 12, 13, 16 and 17

The thiadiazolopyrimidine derivative **3** (0.89 g, 5 mmol) was dissolved in 20 ml pyridine, followed by the addition of the appropriate α-aminoazole reagent (5 mmol) (namely, 5-aminotetrazole, 3-amino-1,2,4-triazole, 5-amino-3-methylpyrazole, 3,5-diamino-4-arylazopyrazole and/or 5-amino-4-arylazopyrazol-3-ol). The mixture was refluxed for 4 h and then diluted with 40 ml of cold water. The solid was collected and recrystallized in an EtOH/DMF mixture (1:1) to produce the tetracyclic compounds **11**, **12**, **13**, **16** and **17**, respectively.

#### 6-Amino-5H-[1,3,4]thiadiazolo[2′,3′:2,3]pyrimido[4,5-d]tetrazolo[1,5-a]pyrimidin-5-one (11)

Yield 57%, m.p. = 274–275 °C. IR: 3341, 3283, 3190 (NH_2_ and N–H), 1645 cm^−1^ (C=O). ^1^H NMR: δ 8.38 (s, 1H, thiadiazole-H), 9.08 ppm (s, 2H). ^13^C NMR: δ 89.36, 143.29, 151.18, 154.50, 161.07, 162.74, 164.91 ppm. MS: m/z (%) = 261 (M^+^, 41.20). Anal. Calcd. for C_7_H_3_N_9_OS (261.02): C, 32.19; H, 1.16; N, 48.26%. Found: C, 32.03; H, 1.18; N, 48.35%.

#### 6-Amino-5H-[1,2,4]triazolo[4′,3′:1,2]pyrimido[5,4-e][1,3,4]thiadiazolo[3,2-a]pyrimidin-5-one (12)

Yield 66%, m.p. = 290–291 °C. IR: 3343, 3270, 3211 (NH_2_ and N–H), 1648 cm^−1^ (C=O). ^1^H NMR: δ 8.37 (s, 1H, thiadiazole-H), 8.61 (s, 1H, triazole-H), 9.16 ppm (s, 2H). ^13^C NMR: δ 91.05, 135.92, 143.63, 148.92, 156.18, 161.84, 162.71, 164.57 ppm. MS: m/z (%) = 260 (M^+^, 73.51). Anal. Calcd. for C_8_H_4_N_8_OS (260.02): C, 36.92; H, 1.55; N, 43.06%. Found: C, 36.80; H, 1.59; N, 43.14%.

#### 6-Amino-9-methyl-5H-pyrazolo[1′,5′:1,2]pyrimido[5,4-e][1,3,4]thiadiazolo[3,2-a]pyrimidin-5-one (13)

Yield 72%, m.p. = 304–306 °C. IR: 3366, 3280, 3178 (NH_2_ and N–H), 1655 cm^−1^ (C=O). ^1^H NMR: δ 2.36 (s, 3H), 6.48 (s, 1H, pyrazole-H), 8.38 (s, 1H, thiadiazole-H), 9.25 ppm (s, 2H). ^13^C NMR: δ 13.53, 92.16, 98.42, 142.87, 146.70, 154.06, 157.35, 160.58, 162.50, 163.73 ppm. MS: m/z (%) = 273 (M^+^, 90.32). Anal. Calcd. for C_10_H_7_N_7_OS (273.04): C, 43.95; H, 2.58; N, 35.88%. Found: C, 44.14; H, 2.51; N, 35.76%.

#### 6,9-Diamino-10-(4-methylphenylazo)-5H-pyrazolo[1′,5′:1,2]pyrimido[5,4-e][1,3,4]thiadiazolo[3,2-a]pyrimidin-5-one (16a)

Yield 75%, m.p. > 320 °C. IR: 3402, 3370, 3277, 3205 (NH_2_ and N–H), 1644 cm^−1^ (C=O). ^1^H NMR: δ 2.32 (s, 3H), 7.36 (d, *J* = 8.50 Hz, 2H), 7.52 (s, 2H), 7.77 (d, *J* = 8.50 Hz, 2H), 8.36 (s, 1H, thiadiazole-H), 8.89 ppm (s, 2H). ^13^C NMR: δ 21.18, 90.49, 100.40, 126.65 (2C), 129.79 (2C), 135.27, 139.38, 141.88, 144.02, 149.76, 157.22, 162.31, 164.80, 166.11 ppm. MS: m/z (%) = 392 (M^+^, 57.30). Anal. Calcd. for C_16_H_12_N_10_OS (392.09): C, 48.97; H, 3.08; N, 35.70%. Found: C, 48.65; H, 3.17; N, 35.84%.

#### 6,9-Diamino-10-(4-methoxyphenylazo)-5H-pyrazolo[1′,5′:1,2]pyrimido[5,4-e][1,3,4]thiadiazolo[3,2-a]pyrimidin-5-one (16b)

Yield 70%, m.p. > 320 °C. IR: 3414, 3361, 3286, 3221 (NH_2_ and N–H), 1648 cm^−1^ (C=O). ^1^H NMR: δ 3.78 (s, 3H), 7.05 (d, *J* = 9.00 Hz, 2H), 7.46 (s, 2H), 7.81 (d, *J* = 9.00 Hz, 2H), 8.35 (s, 1H, thiadiazole-H), 8.84 ppm (s, 2H). ^13^C NMR: δ 55.74, 88.65, 97.88, 114.52 (2C), 128.11 (2C), 132.63, 140.37, 143.18, 150.26, 157.76, 159.88, 161.17, 164.92, 167.41 ppm. MS: m/z (%) = 408 (M^+^, 42.88). Anal. Calcd. for C_16_H_12_N_10_O_2_S (408.09): C, 47.06; H, 2.96; N, 34.30%. Found: C, 46.90; H, 2.90; N, 34.19%.

#### 6-Amino-9-hydroxy-10-(4-methylphenylazo)-5H-pyrazolo[1′,5′:1,2]pyrimido[5,4-e][1,3,4]thiadiazolo[3,2-a]pyrimidin-5-one (17a)

Yield 59%, m.p. = 264–265 °C. IR: 3370, 3253, 3180 (NH_2_ and N–H), 1655 cm^−1^ (C=O). ^1^H NMR: δ 2.32 (s, 3H), 7.35 (d, *J* = 8.50 Hz, 2H), 7.74 (d, *J* = 8.50 Hz, 2H), 8.33 (s, 1H, thiadiazole-H), 8.97 (s, 2H), 11.78 ppm (s, 1H). ^13^C NMR: δ 21.17, 91.08, 103.75, 126.79 (2C), 129.73 (2C), 136.06, 139.23, 143.81, 145.45, 148.94, 153.98, 162.46, 164.57, 166.15 ppm. MS: m/z (%) = 393 (M^+^, 48.29). Anal. Calcd. for C_16_H_11_N_9_O_2_S (393.08): C, 48.85; H, 2.82; N, 32.05%. Found: C, 48.98; H, 2.86; N, 32.12%.

#### 6-Amino-9-hydroxy-10-(4-methoxyphenylazo)-5H-pyrazolo[1′,5′:1,2]pyrimido[5,4-e][1,3,4]thiadiazolo[3,2-a]pyrimidin-5-one (17b)

Yield 64%, m.p. = 280–281 °C. IR: 3351, 3269, 3172 (NH_2_ and N–H), 1658 cm^−1^ (C=O). ^1^H NMR: δ 3.78 (s, 3H), 7.06 (d, *J* = 9.00 Hz, 2H), 7.78 (d, *J* = 9.00 Hz, 2H), 8.32 (s, 1H, thiadiazole-H), 8.94 (s, 2H), 11.90 ppm (s, 1H). ^13^C NMR: δ 55.71, 90.84, 102.60, 114.58 (2C), 128.47 (2C), 131.61, 143.12, 145.04, 152.45, 158.38, 159.73, 163.10, 164.75, 166.69 ppm. MS: m/z (%) = 409 (M^+^, 57.04). Anal. Calcd. for C_16_H_11_N_9_O_3_S (409.07): C, 46.94; H, 2.71; N, 30.79%. Found: C, 46.81; H, 2.78; N, 30.70%.

### Biology

The biological examination procedures are fully deliberated in the supplementary material.

#### Antibacterial and antifungal activity

The synthesized thiadiazolopyrimidine-based compounds were evaluated and adopted for antibacterial and antifungal effectiveness using the approved procedure^38,39^.

#### Antiquorum-sensing analysis

The anti-QS inefficiency was evaluated using the previous technique mentioned in the literature^40^.

#### MTT cytotoxicity assay

The synthesized compounds were subjected to the MTT cytotoxicity assay on the basis of the cited literature^41^.

#### In silico docking

The in silico study was conducted to debate the mode of binding in the prepared thiadiazolopyrimidine polycyclic compounds with the crystal structure potent inhibitors of NUDT5 silence hormone signaling in breast cancer (PDB Code-5NQR)^37^. The PDB format of the protein was designated from the homepage of the protein data bank and applied using the MOE v10.2015.10 program.

## Supplementary Information

Supplementary Information.
