# Supplementary material for: Synthesis and biological screening of new thiadiazolopyrimidine-based polycyclic compounds
Source: Sci Rep. 2021 Aug 3;11:15750. doi: 10.1038/s41598-021-95241-x (PMC8333053; doi:10.1038/s41598-021-95241-x)
Supplement: Supplementary file 1 — Supplementary Information. [file 41598_2021_95241_MOESM1_ESM.docx]

**Synthesis and Biological Screening of New Thiadiazolopyrimidine-based Polycyclic Compounds**

**Alaa M. Alqahtani**

Pharmaceutical Chemistry Department, College of Pharmacy, Umm Al-Qura University, 21955 Makkah, Saudi Arabia

Email address: [amqahtani@uqu.edu.sa](mailto:amqahtani@uqu.edu.sa)

**Biological Procedures**

**Antibacterial screening (determination of minimal inhibitory concentration) (MIC, µg/mL).**

Minimal inhibitory concentrations (MICs) were determined by serial dilution technique using 96-multiwell microtiter plates. The investigated compounds were dissolved in DMSO 100% to prepare stock solutions of 5000 µg/mL. Two-fold serial dilutions of the dissolved compounds were performed in LB broth providing eight different concentrations (1250, 625, 312.5, 156, 78, 39, 19.5 µg/mL). Overnight cultures of microorganisms were diluted to 1 ×10^6^ CFU/mL in LB broth, and 20 mL of the diluted cultures were added to the test solutions (50 mL) in the wells (one well per concentration per bacterial strain). The plates were incubated at 37°C for 24 h. MIC was detected visually as the least concentration inhibiting bacterial growth (no turbidity) and the activity of the tested compounds was estimated in comparison to ampicillin.

**Antifungal screening (determination of minimal inhibitory concentration) (MIC, µg/mL).**

Minimal inhibitory concentrations (MICs) were determined by serial dilution technique using 96-multiwell microtiter plates. The investigated compounds were dissolved in DMSO 100% to prepare stock solutions of 5000 µg/mL. Two-fold serial dilutions of the dissolved compounds were performed in glucose minimal medium providing eight different concentrations (1250, 625, 312.5, 156, 78, 39, 19.5 µg/mL). The microorganisms were diluted to 1 ×10^3^ SFU/mL in glucose minimal medium, and 20 µL of the diluted culture was added to the test solutions (50 µL) in the wells (one well per concentration). The plates were incubated at 30°C for 48 h. MIC was detected visually as the least concentration inhibiting fungal growth (no turbidity) and the activity of the tested compounds was estimated in comparison to fluconazole.

**Antiquorum-sensing assay.**

The culture was prepared by growing *Ch. violaceum* ATCC 12472 in LB broth and incubated for 16–18 h in an orbital incubator running at 28°C and 150 rpm. The culture was then adjusted to 0.5 McFarland standard (ca. 1 × 10^6^ CFU/mL). *Ch. violaceum* (50 µL) was inoculated into LB agar (50 mL), poured into plates, and solidified. Wells were made in LB agar medium using cork borer. The tested compounds were dissolved in DMSO 100% in Eppendorf tubes for final concentration of 5 mg/mL, and 50 µL of the test solution was applied into the wells. The positive control (catechin) was also added at the same concentration and volume to each plate. In addition, DMSO (control solvent) was added to each plate. Plates were incubated at 30°C for 48 h to check the inhibition of pigment production around the wells. Bacterial growth inhibition would result in a clear halo around the disc, while a positive quorum sensing inhibition is exhibited by a turbid halo harboring pigmentless bacterial cells of *Ch. violaceum* ATCC 12472 monitor strain. Bacterial growth inhibition by the tested compounds was measured as radius (r_1_) in mm, while both growth and pigment inhibition was measured as radius (r_2_) in mm. The pigment inhibition (QS inhibition) was determined by subtracting bacterial growth inhibition (r_1_) from the total radius (r_2_); thus, QS inhibition = (r_2_ - r_1_) in mm.

**MTT cytotoxicity assay:** The technical method of MTT assay depends on the reduction of yellow 3-(4,5-methyl-2-thiazolyl)-2,5-diphenyl-2*H*-tetrazolium bromide (MTT) into purple formazan product, mainly by mitochondrial reductase activity inside the living cells. The cells used in cytotoxicity assay were cultured in RPMI 1640 medium supplemented with 10% fetal calf serum. Cells suspended in the medium (2×10^4^ cells/mL) were plated in 96-well culture plates and incubated at 37 °C in a 5% CO_2_ incubator for 12 hours. The tested sample (2 μL) was added to the cells (2×10^4^) in 96-well plates and cultured at 37 °C for 3 days. The cultured cells were mixed with 20 μL of MTT solution and incubated for 4 hours at 37 °C. The supernatant was carefully removed from each well, the formazan crystals were dissolved after addition of DMSO (100 μL) for each well to form the cellular reduction of MTT. After mixing with a mechanical plate mixer, the absorbance of each well was measured by a microplate reader using a test wavelength of 570 nm. The results were expressed as the IC_50_ values, which inducing 50% inhibition of cell growth of the treated cells when compared to the growth of control cells.

**Bleomycin-dependent DNA damage experiment:** To a mixture of 0.5 mg / mL of DNA, 5 mM magnesium chloride, 50 mM ferric chloride and 0.05 mg / mL bleomycin sulfate was prepared. The test sample was prepared at a concentration of 0.1 mg / mL and added to the mixture. After incubation of the mixture for one hour at 37 °C, 0.05 mL of EDTA (0.1 M) was added to terminate the reaction. In the next step, 0.5 mL of TBA (1% w / v) and 0.5 mL of HCl (25% v / v) were added for colour development and the mixture was heated for 10 minutes at 80 °C. The mixture was centrifuged and the absorbance expressed, the amount of damage to the DNA was measured at λ = 532 nm. L-Ascorbic acid (0.24 mM) was used as a positive control.

***In silico* *docking***

| 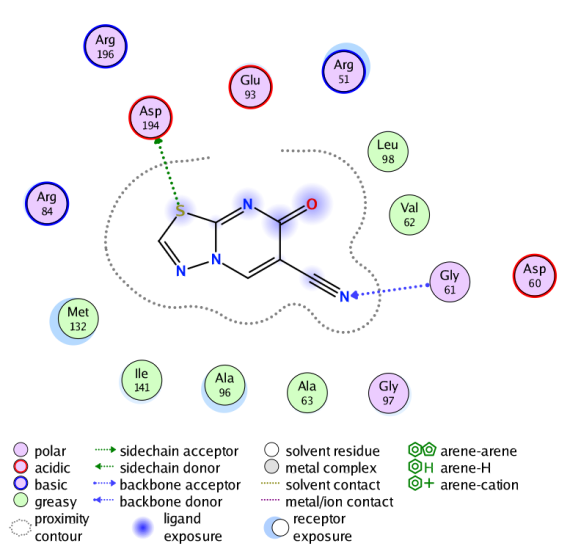  2D | 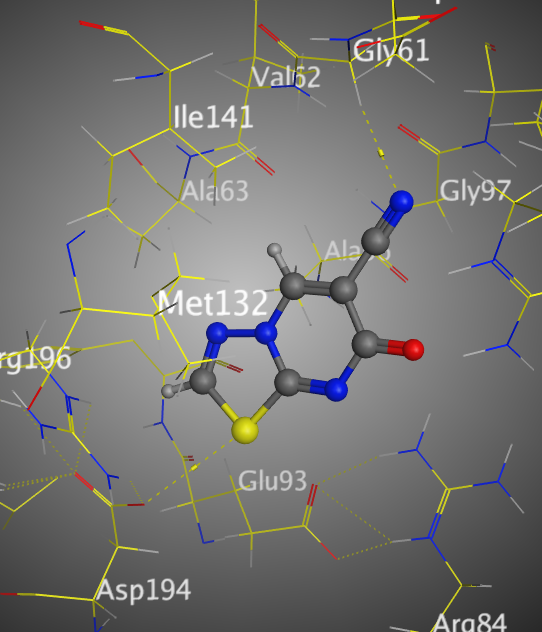  3D |
| --- | --- |

Figure S1. The binding interaction of **3** with (PDB ID: 5NQR).

| 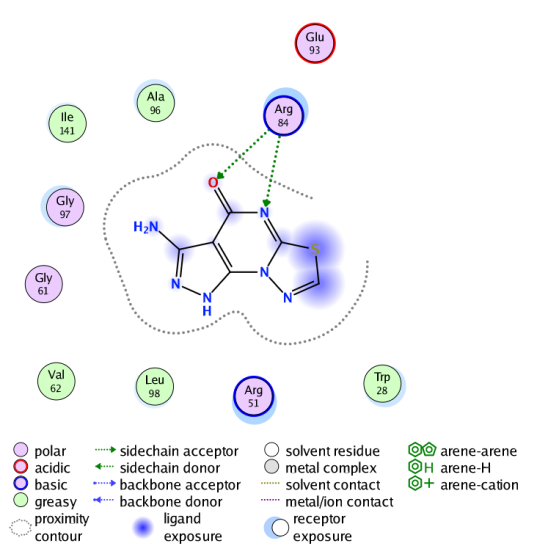  2D | 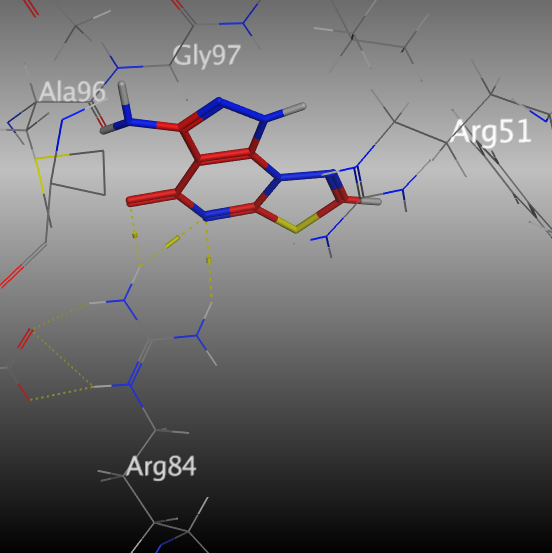  3D |
| --- | --- |

Figure S2. The binding interaction of **4** with (PDB ID: 5NQR).

| 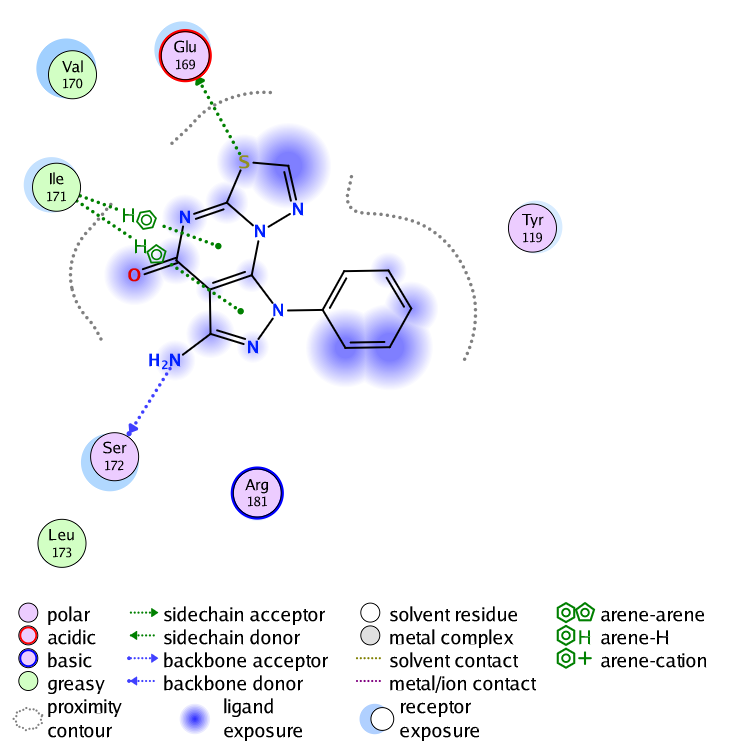  2D | 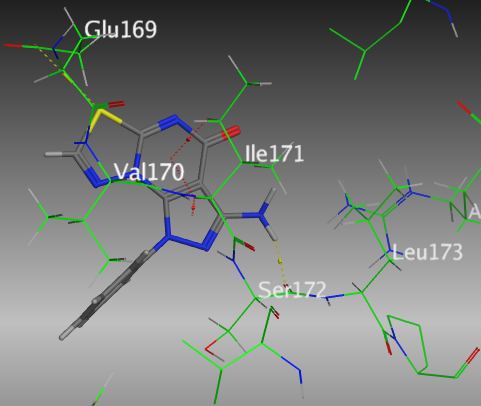  3D |
| --- | --- |

Figure S3. The binding interaction of **5** with (PDB ID: 5NQR).

| **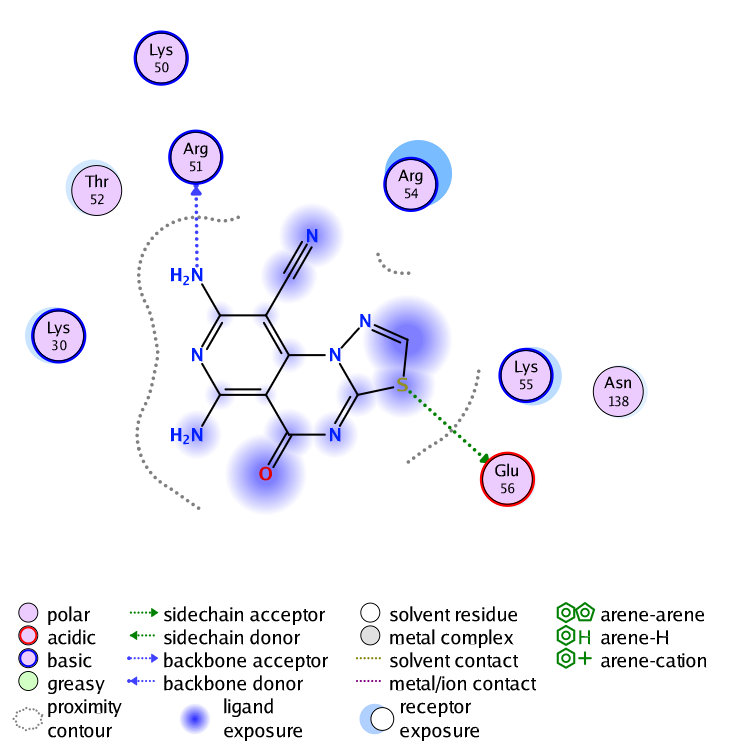**  2D | 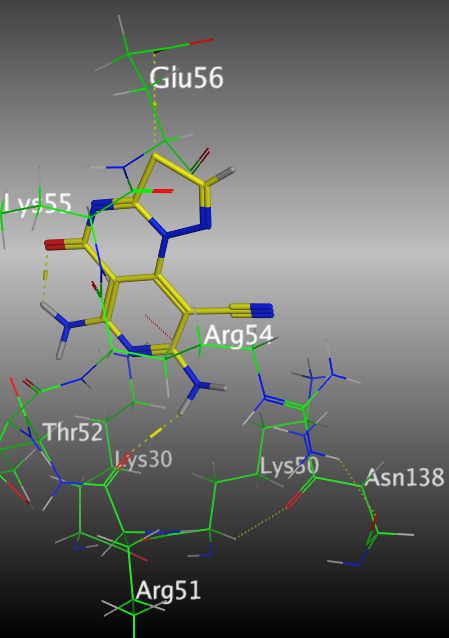  3D |
| --- | --- |

Figure S4. The binding interaction of **6** with (PDB ID: 5NQR).

| 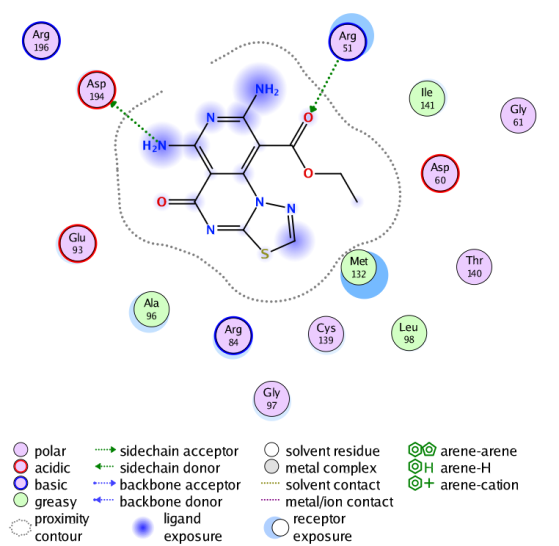  2D | 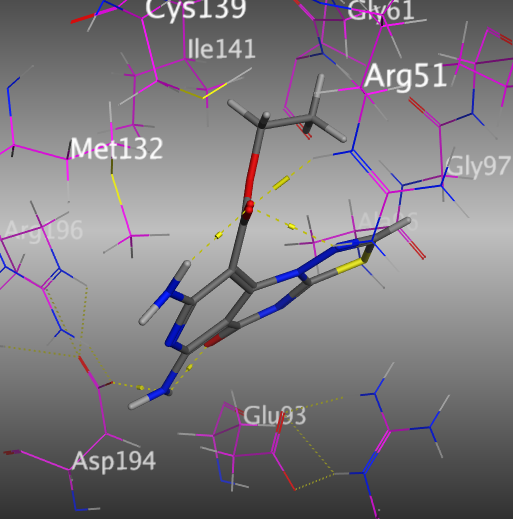  3D |
| --- | --- |

Figure S5. The binding interaction of **7** with (PDB ID: 5NQR).

| 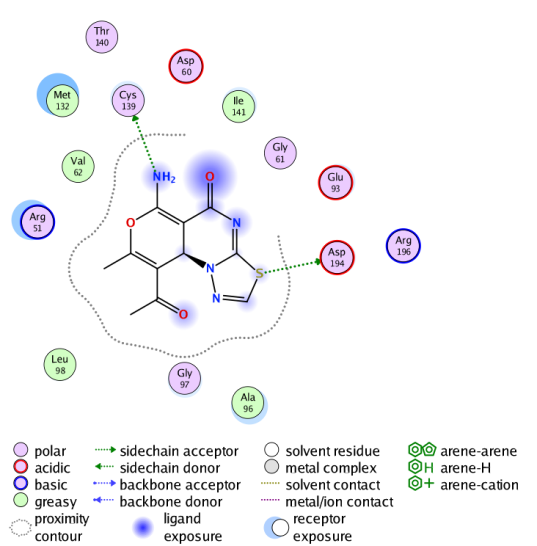  2D | 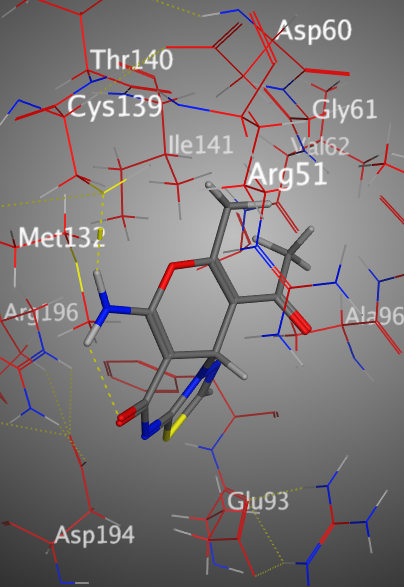  3D |
| --- | --- |

Figure S6. The interaction of **8a** with (PDB ID: 5NQR).

| 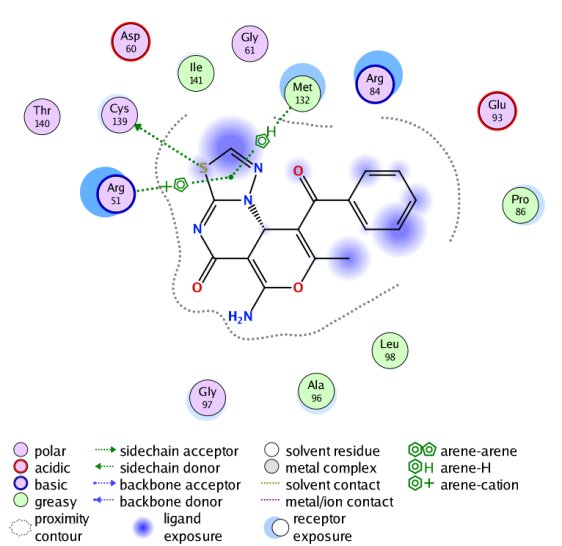  2D | 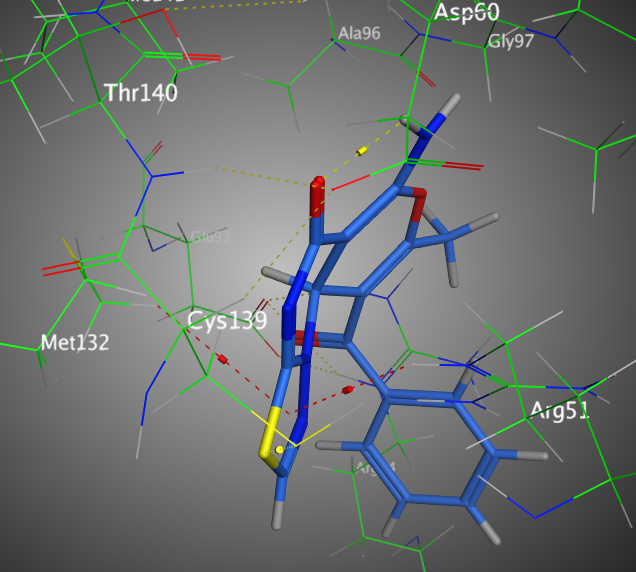  3D |
| --- | --- |

Figure S7. The binding interaction of **8b** with (PDB ID: 5NQR).

| 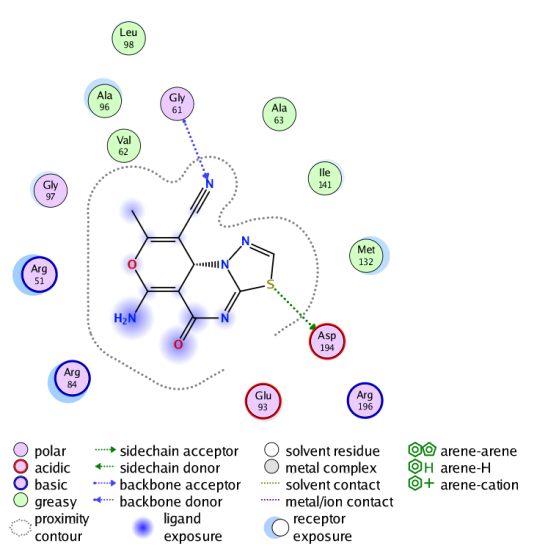  2D | 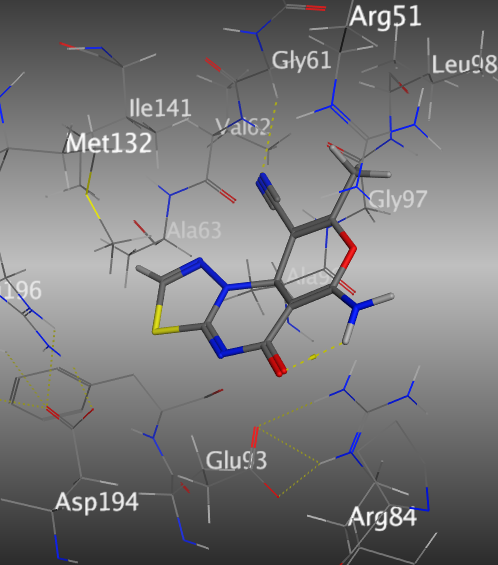  3D |
| --- | --- |

Figure S8. The binding interaction of **9a** with (PDB ID: 5NQR).

| 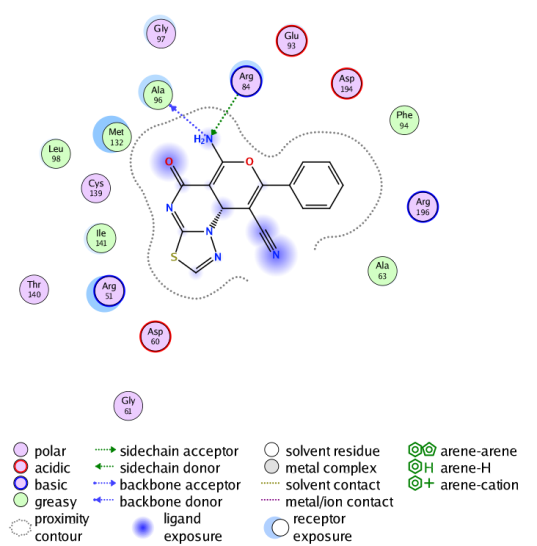  2D | 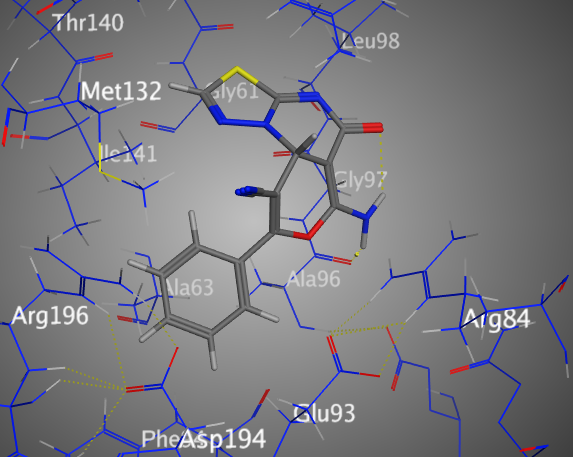  3D |
| --- | --- |

Figure S9. The binding interaction of **9b** with (PDB ID: 5NQR).

| 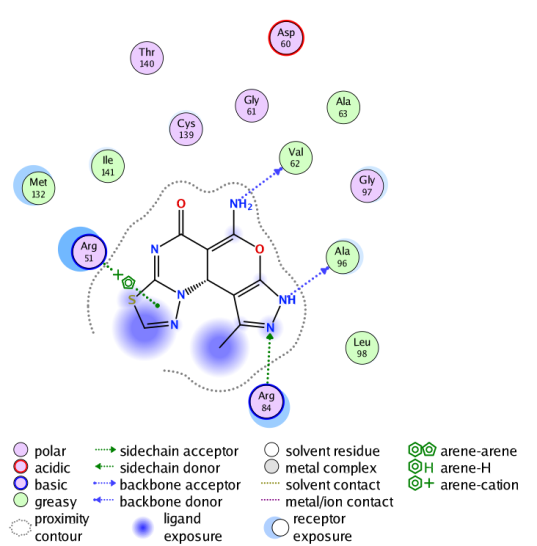  2D | 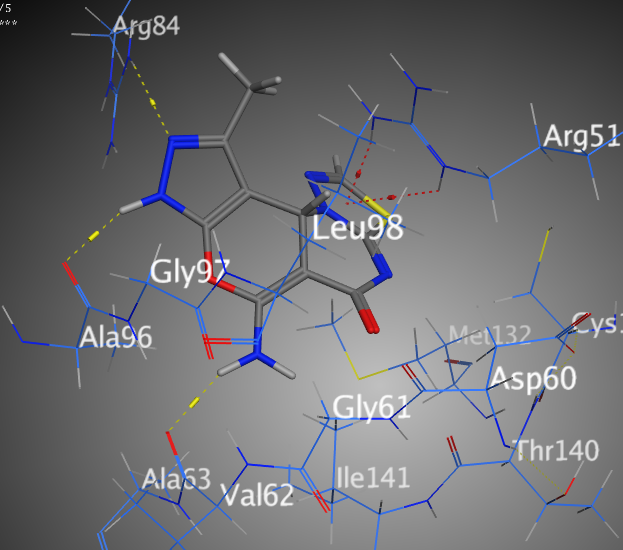  3D |
| --- | --- |

Figure S10. The binding interaction of **10a** with (PDB ID: 5NQR).

| 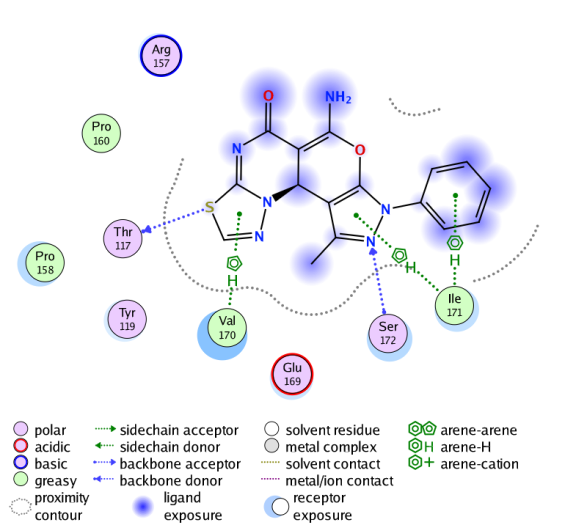  2D | 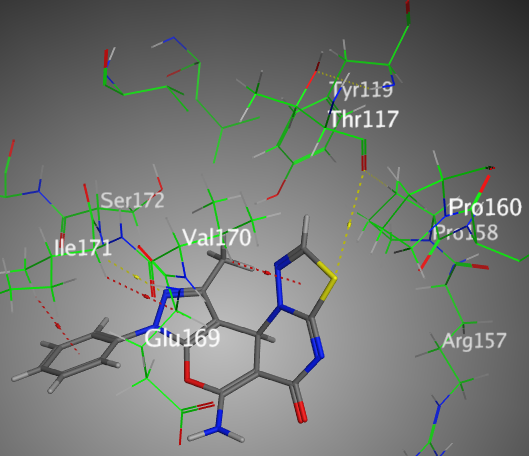  3D |
| --- | --- |

Figure S11. The binding interaction of **10b** with (PDB ID: 5NQR).

| 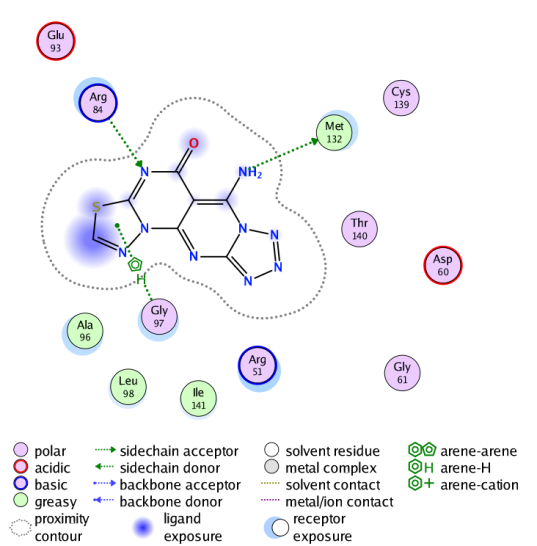  2D | 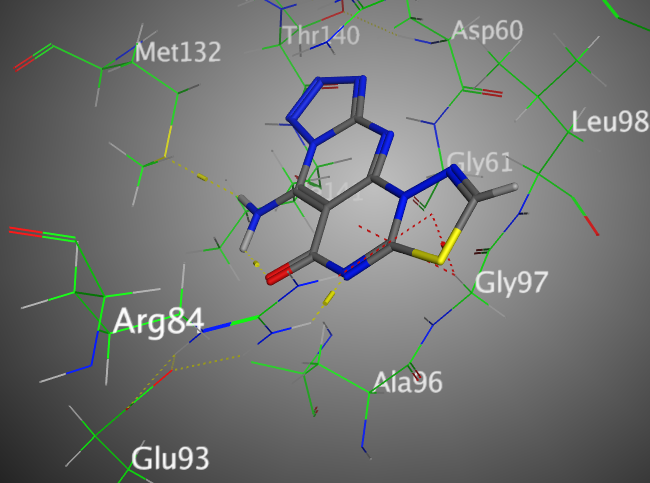  3D |
| --- | --- |

Figure S12. The binding interaction of **11** with (PDB ID: 5NQR).

| 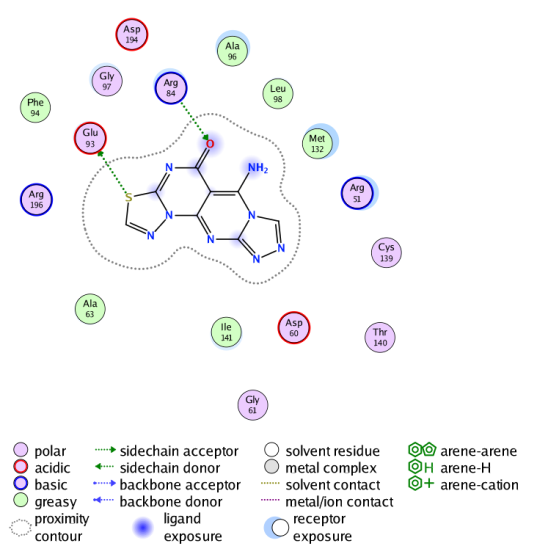  2D | 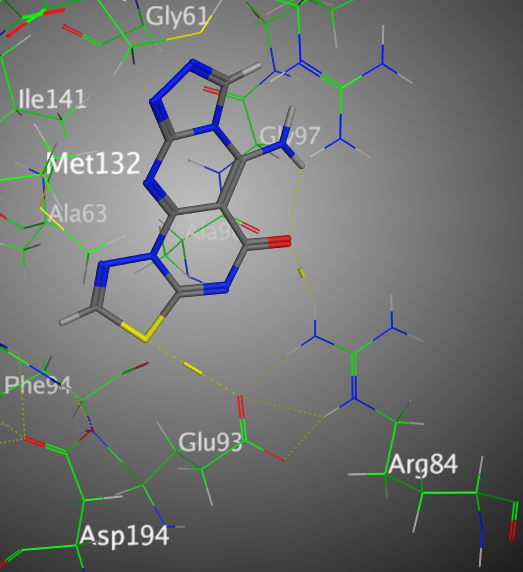  3D |
| --- | --- |

Figure S13. The binding interaction of **12** with (PDB ID: 5NQR).

| 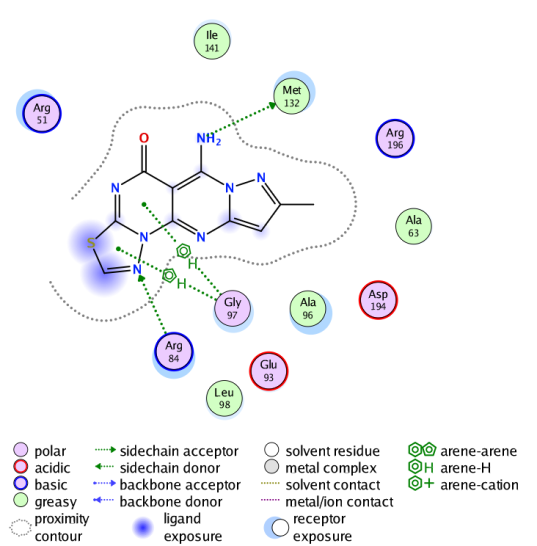  2D | 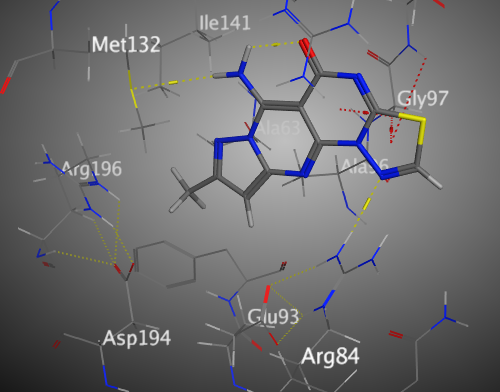  3D |
| --- | --- |

Figure S14. The binding interaction of **13** with (PDB ID: 5NQR).

| 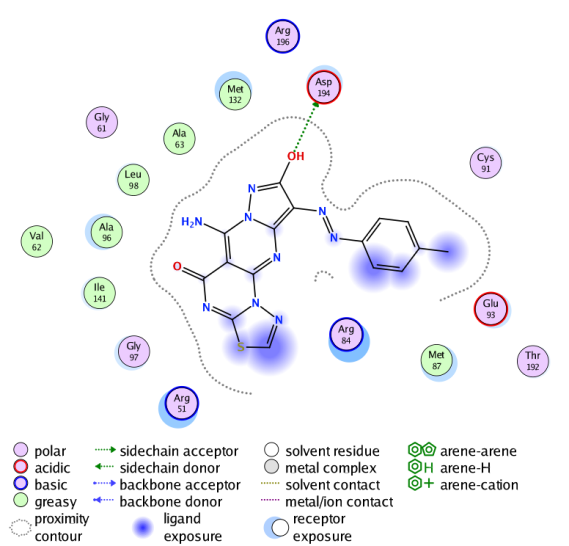  2D | 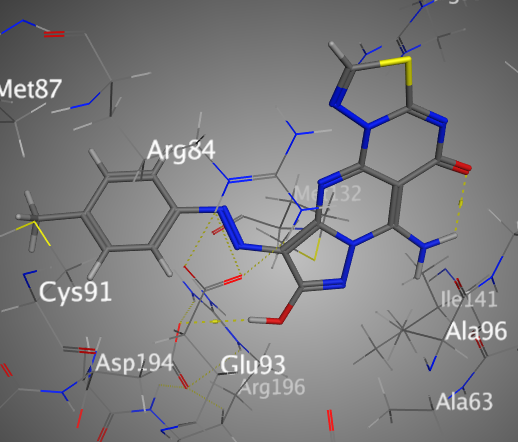  3D |
| --- | --- |

Figure S15. The binding interaction of **17a** with (PDB ID: 5NQR).

| 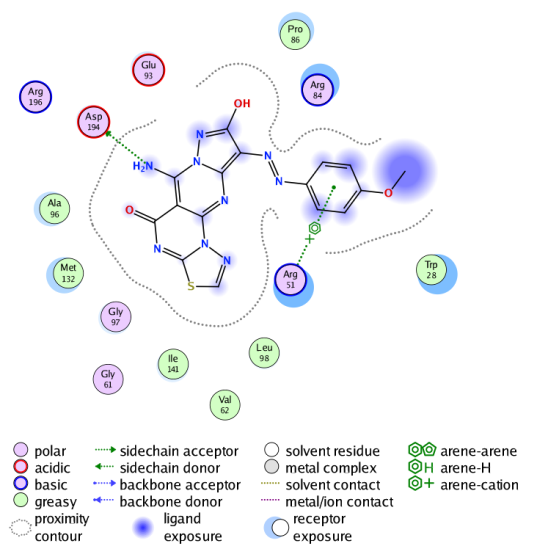  2D | 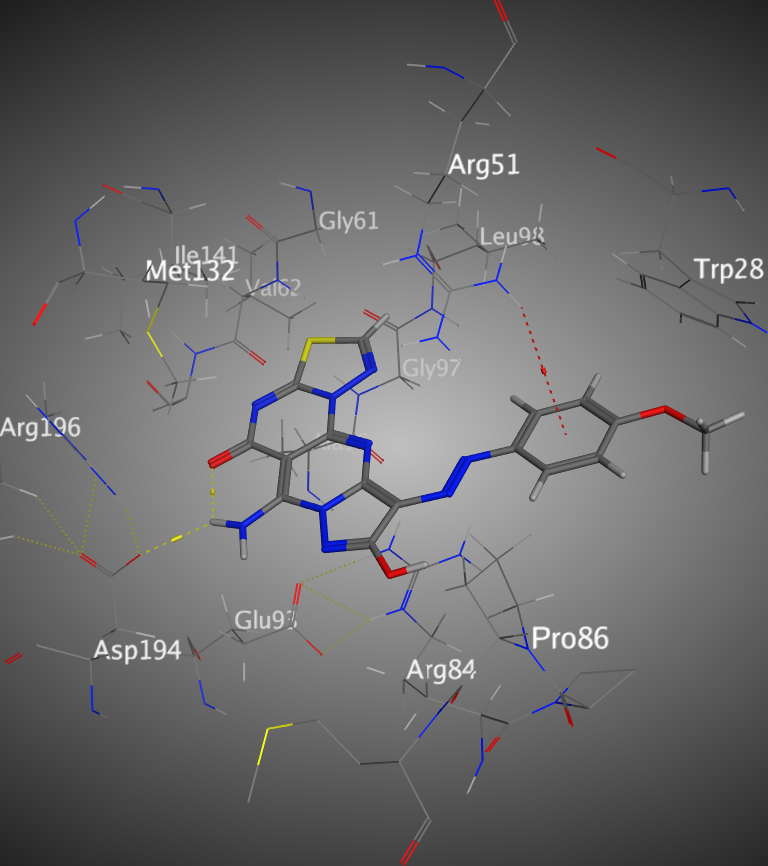  3D |
| --- | --- |

Figure S16. The binding interaction of **17b** with (PDB ID: 5NQR).

| 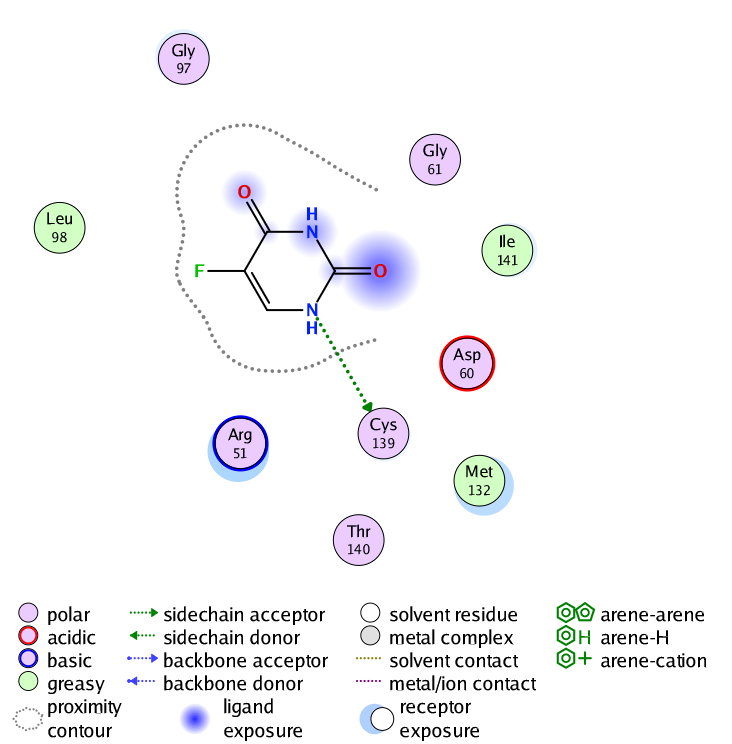  2D | 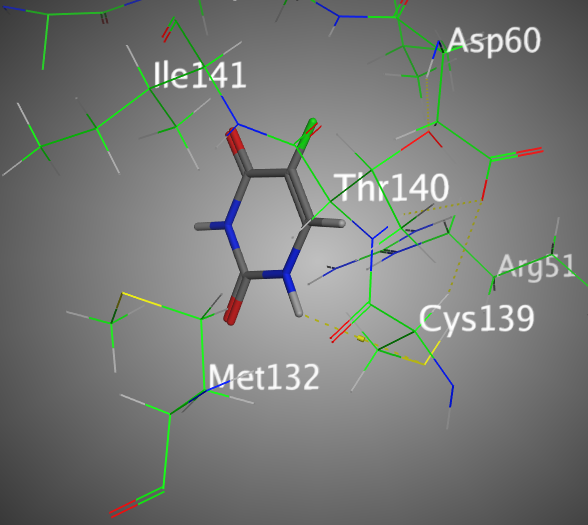  3D |
| --- | --- |

Figure S17. The binding interaction of **5-Fluorouracil** with (PDB ID: 5NQR).

Figure (S18): ^1^H NMR spectrum of compound (**2**)

Figure (S19): ^1^H NMR spectrum of compound (**3**)

Figure (S20): ^13^C NMR spectrum of compound (**3**)

Figure (S21): ^1^H NMR spectrum of compound (**4**)

Figure (S22): ^13^C NMR spectrum of compound (**4**)

Figure (S23): ^1^H NMR spectrum of compound (**5**)

Figure (S24): ^13^C NMR spectrum of compound (**5**)

Figure (S25): ^1^H NMR spectrum of compound (**6**)


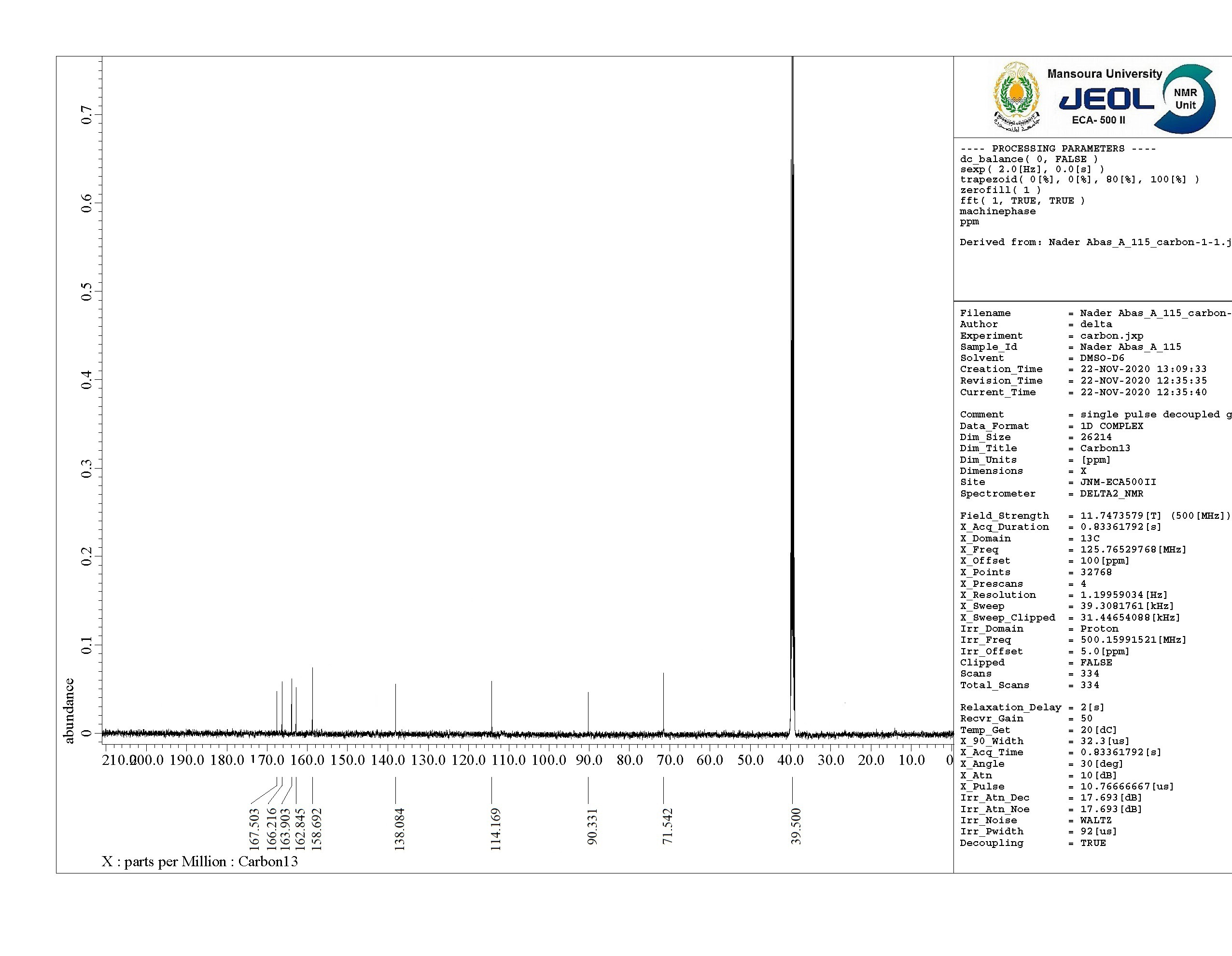

Figure (S26): ^13^C NMR spectrum of compound (**6**)

Figure (S27): ^1^H NMR spectrum of compound (**7**)


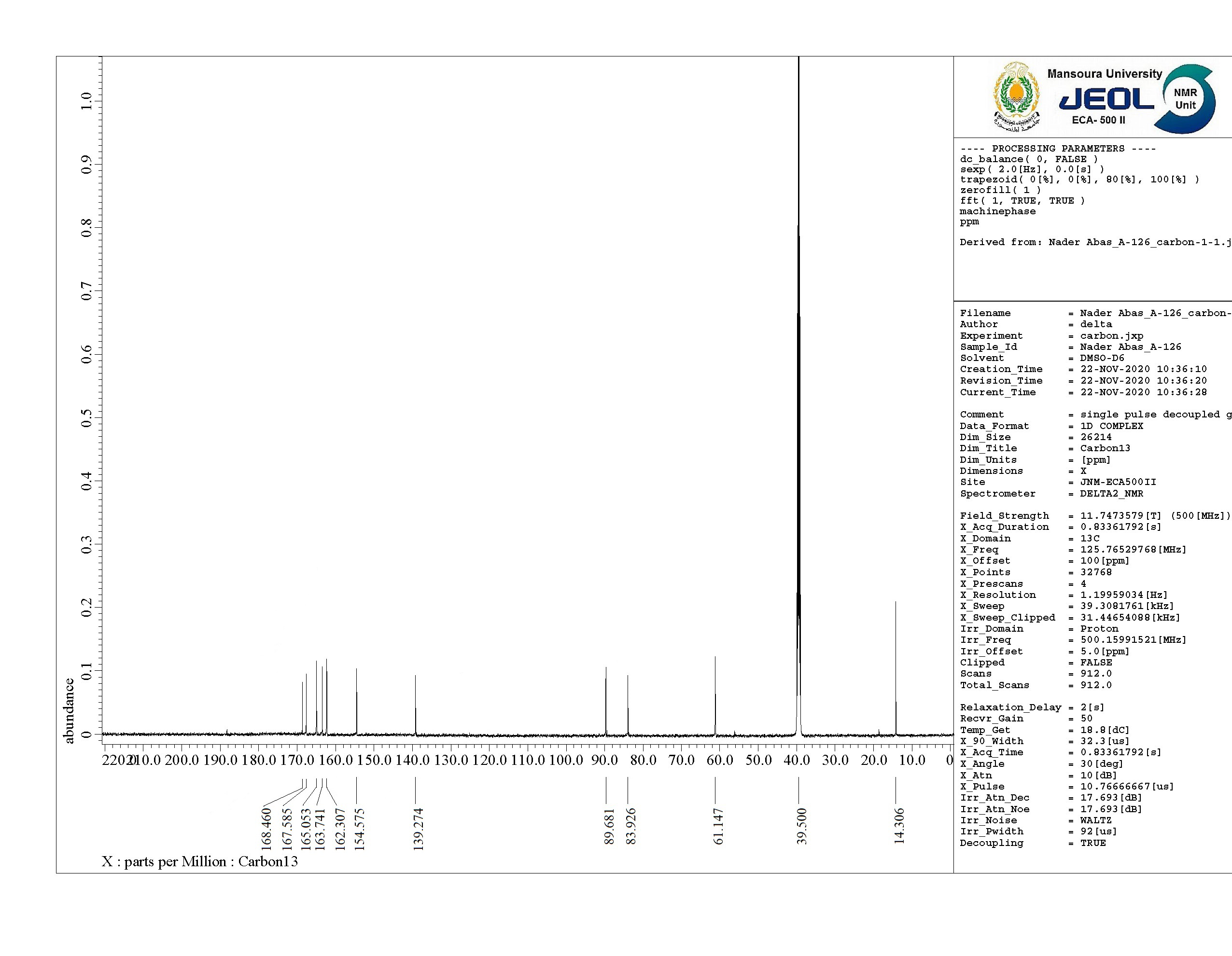

Figure (S28): ^13^C NMR spectrum of compound (**7**)

Figure (S29): ^1^H NMR spectrum of compound (**8a**)

Figure (S30): ^13^C NMR spectrum of compound (**8a**)

Figure (S31): ^1^H NMR spectrum of compound (**8b**)

Figure (S32): ^13^C NMR spectrum of compound (**8b**)

Figure (S33): ^1^H NMR spectrum of compound (**9a**)

Figure (S34): ^13^C NMR spectrum of compound (**9a**)

Figure (S35): ^1^H NMR spectrum of compound (**9b**)

Figure (S36): ^13^C NMR spectrum of compound (**9b**)

Figure (S37): ^1^H NMR spectrum of compound (**10a**)

Figure (S38): ^13^C NMR spectrum of compound (**10a**)

Figure (S39): ^1^H NMR spectrum of compound (**10b**)

Figure (S40): ^13^C NMR spectrum of compound (**10b**)

Figure (S41): ^1^H NMR spectrum of compound (**11**)

Figure (S42): ^13^C NMR spectrum of compound (**11**)

Figure (S43): ^1^H NMR spectrum of compound (**12**)

Figure (S44): ^13^C NMR spectrum of compound (**12**)

Figure (S45): ^1^H NMR spectrum of compound (**13**)

Figure (S46): ^13^C NMR spectrum of compound (**13**)

Figure (S47): ^1^H NMR spectrum of compound (**16a**)

Figure (S48): ^13^C NMR spectrum of compound (**16a**)

Figure (S49): ^1^H NMR spectrum of compound (**16b**)

Figure (S50): ^13^C NMR spectrum of compound (**16b**)

Figure (S51): ^1^H NMR spectrum of compound (**17a**)

Figure (S52): ^13^C NMR spectrum of compound (**17a**)

Figure (S53): ^1^H NMR spectrum of compound (**17b**)

Figure (S54): ^13^C NMR spectrum of compound (**17b**)
